# BODIPY Photocage‐Based Injectable Hydrogel for Light‐Controlled Nanoparticle Release

**DOI:** 10.1002/smll.74162

**Published:** 2026-07-01

**Authors:** Baihao Shao, David J. Peeler, Thomas F. F. Fernandez Debets, Jonathan P. Wojciechowski, Yue Shao, Yuxi Cheng, Kun Zhou, Robin J. Shattock, Molly M. Stevens

**Affiliations:** ^1^ Department of Physiology, Anatomy and Genetics Department of Engineering Science and Kavli Institute for Nanoscience Discovery University of Oxford Oxford UK; ^2^ Department of Materials Department of Bioengineering Institute of Biomedical Engineering Imperial College London London UK; ^3^ Department of Infectious Disease Imperial College London London UK

**Keywords:** click chemistry, controlled release, injectable hydrogel, photocage

## Abstract

Light‐controlled release of therapeutics holds great promise for improving patient compliance with treatment plans against many leading diseases and could contribute to a better quality of life for people suffering from chronic conditions. However, insufficient tissue penetration of light largely limits the in vivo applications of photoresponsive therapeutics. With the aim to circumvent this limitation and to work within tissue depths that are pragmatically accessible to light, we developed an injectable and photodegradable hydrogel that could enable controlled drug delivery in subcutaneous tissue. In this work, we describe a hydrogel formulation consisting of a 2‐arm photocage bearing azide and a BCN‐terminated 4‐arm PEG. Gelation takes place in minutes upon mixing the two components via strain‐promoted azide‐alkyne cycloaddition (SPAAC). Both gel precursors are made water‐soluble and exhibit low viscosity prior to complete gelation, and the pre‐gel is easily injectable through 23G needles. Photocage photocleavage and consequent gel degradation could be precisely controlled with green light irradiation. We further showed that nanoparticles can be successfully encapsulated into the gel and subsequently photo‐released. The full operation process was demonstrated in an ex vivo porcine model and the acute biocompatibility of gel injection and degradation was evaluated in healthy human skin cultured ex vivo.

## Graphical abstract

This work presents an injectable hydrogel formulation which undergoes efficient photodegradation under green light irradiation. The gel can be easily injected to subcutaneous tissue through 23G needles, co‐encapsulate and photo‐release nanoparticles as well as exhibits excellent biocompatibility, making it promising for the development of light‐controlled subcutaneous delivery systems in vivo.

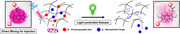

## 1 Introduction

Light is a non‐invasive and biocompatible external stimulus. When coupled with a light‐responsive system, it can be used to enable spatiotemporal control over protein activation or inhibition [1, 2], transcription and translation [3], cell patterning and differentiation [4, 5], and therapeutic release [6] among other dynamic functions. Unfortunately, the limited tissue penetration of light presents a daunting barrier that largely hinders the translation of light‐activated biochemical tools in vivo. Recent efforts in red‐shifting the activation wavelength of photoresponsive molecules and materials [7, 8], developing biocompatible optoelectronic devices [9, 10] and applying these tools with endoscopic procedures [11, 12] have tremendously expanded the scope and utility of photoresponsive systems in biomedical applications. These strategies, however, inevitably increase complexity in the systems, requiring synthetically challenging light‐activated motifs or surgical interventions to ensure in vivo efficacy. Subcutaneous administration is one of the most accessible delivery methods in clinical settings attributing to its low cost, ease of operation and excellent patient compliance [13]. Specifically, the hypodermis, where subcutaneous injection normally takes place, is highly vascularized and contains abundant blood and lymphatic capillaries, enabling locally administered therapeutics to enter systemic circulation. Subcutaneous tissue is known to be rich in adipocytes and features reduced perfusion, making it ideal for long‐acting medications. Most importantly, the hypodermis is located on average *ca*. 2.1 mm below the epidermis [14], a depth at which visible light (400–700 nm) could be efficiently introduced to photo‐activate and/or ‐release therapeutics therein [15, 16] with readily accessible photochemical tools and minimal invasive interventions.

**FIGURE 1:**
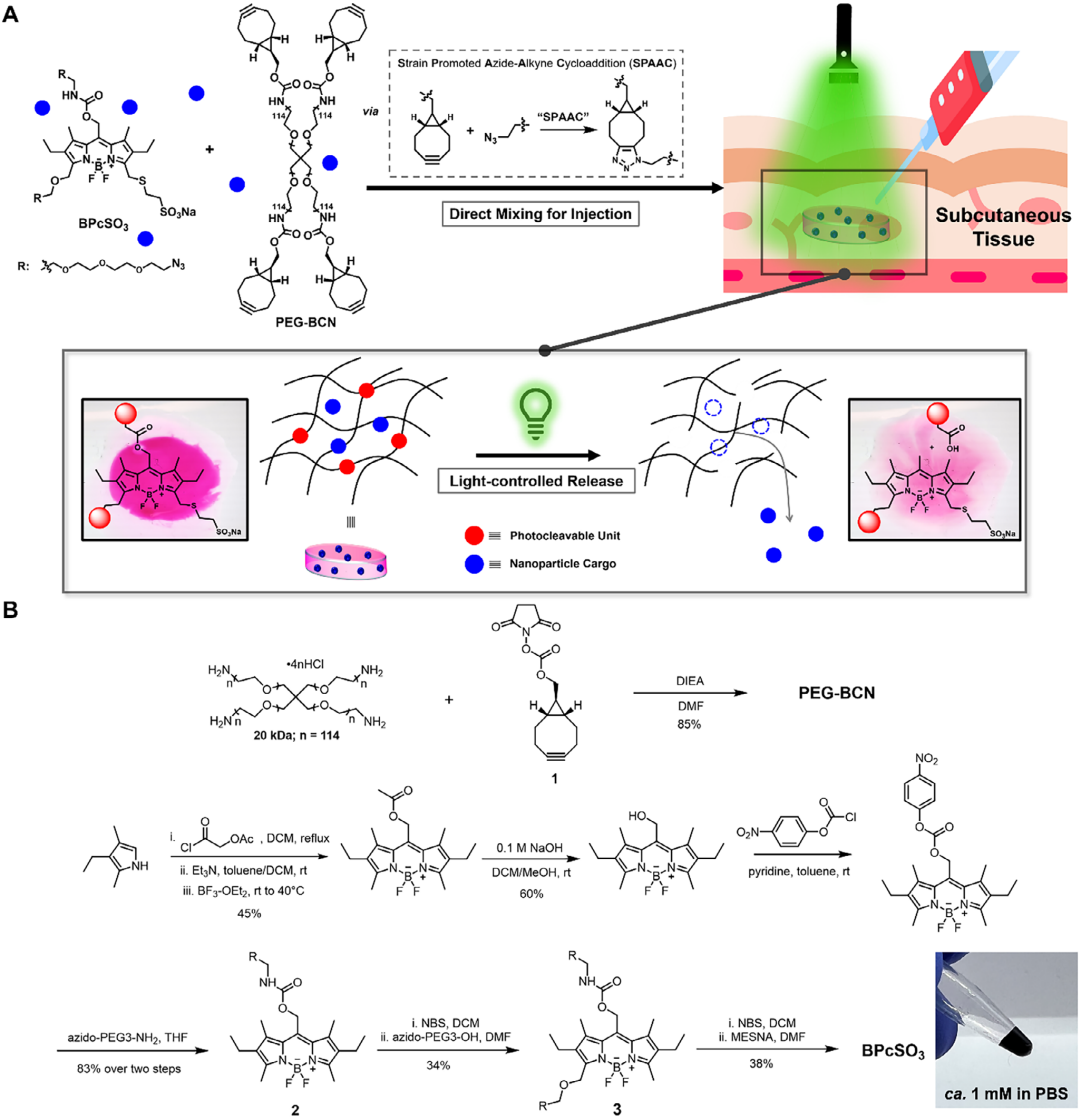
Design rationale of an injectable and photodegradable hydrogel formulation for subcutaneous drug delivery and synthetic procedures towards two hydrogel precursors **PEG‐BCN** and **BPcSO_3_**. (A) Schematic of an injectable hydrogel platform for light‐controlled subcutaneous release. Cargo loading can be achieved through mixing both **BPcSO_3_** and **PEG‐BCN** in the presence of the cargo. The pre‐gel mixture is suitable for direct injection through 21/23G needles and gelation will continue to completion in situ after deposition. Photo‐cleavage of the BPc crosslinkers upon light irradiation results in gel degradation and subsequent cargo release. (B) 20 kDa 4‐arm PEG‐NH_2_ hydrochloride was functionalized with strained alkynes (BCN) in a basic condition, while the water‐soluble **BPcSO_3_** bearing two azido PEG chains was synthesized in six steps. The inset image shows a 1 mM solution of **BPcSO_3_** in PBS.

Hydrogels, a class of soft porous materials made from crosslinked three‐dimensional polymer networks which retain large amounts of water, are utilized in medical supports and replacements, tissue repair and regeneration, and the controlled delivery of small‐molecule and macromolecular therapeutics [17, 18, 19, 20, 21, 22, 23, 24]. The mechanical, chemical and biological properties of hydrogels are highly tunable, with those characterized by shear‐thinning, shape‐memory and in situ‐gelling properties suitable for the development of injectable gel formulations [25, 26]. Compared to implantation, injection is minimally invasive and less resource demanding, thus lowering costs and enhancing patient compliance [27, 28], all of which make an injectable hydrogel an ideal material for subcutaneous drug delivery. Photocleavable compounds, i.e., photocages [29, 30, 31, 32, 33, 34, 35, 36], undergoing one‐way photochemical bond dissociation have been widely utilized to develop photoresponsive hydrogels, enabling a broad spectrum of biomedical applications ranging from controlled release [37, 38, 39, 40, 41, 42, 43] to 4D biomaterial customization [44, 45]. However, few studies have investigated the injectability aspect of photoresponsive hydrogels in the context of subcutaneous drug delivery [46, 47, 48, 49]. In view of the ease of light penetration, subcutaneous deposition and light‐dependent modulation, we focused our attention on developing a hydrogel formulation that is able to encapsulate nanoparticle cargo, to be injected through a syringe, and to degrade photochemically to release the nanoparticles. We reasoned that such a system would enable drug carriers to be activated in the hypodermis for controlled photo‐release by accessible light. Among the photocages developed over the years [29, 30, 31, 32, 33, 34, 35, 36], boron dipyrromethenes (BODIPY) photocages (BPc) deriving from the widely used bioimaging fluorophores, display good biocompatibility, visible‐to‐NIR light activation, high photo‐uncaging efficiency and practical synthetic accessibility [50]. To this end, we report an in situ‐gelling, loadable, injectable and photodegradable hydrogel system, which utilizes a strain‐promoted azide‐alkyne cycloaddition (SPAAC) [51, 52] between a di‐azido BPc linker (**BPcSO_3_**) and a tetra‐armed bicyclononyne (BCN)‐terminated poly(ethylene glycol) macromer (**PEG**‐**BCN**) (Figure 1A). Both **BPcSO_3_** and **PEG**‐**BCN** have reasonable aqueous solubilities (i.e. mM concentrations) such that gelation can occur in PBS (phosphate buffered saline) within a few minutes. Efficient photo‐uncaging of the BPc units under green light irradiation within the gel ensures fast photodegradation of the hydrogel network. The gel degradation process can be photo‐modulated in a precise and non‐invasive manner. Gel formation and photodegradation proceeded in the presence of co‐encapsulated polystyrene nanoparticles and ovalbumin, and the controlled release of the nanoparticles was achieved with minimal ambient release in dark controls. Finally in ex vivo porcine and human skin models, we demonstrated that the gel mixture can be easily loaded into a syringe through aspiration, the pre‐gel solution can be directly injected beneath the skin, gelation will continue to completion in situ, and gel degradation can be triggered by green light irradiation.

## 2 Results and Discussion

### 2.1 Synthesis and Characterization of the Gel Precursors

We started by synthesizing the gel precursors (Figure 1B). Strained alkyne NHS carbonate 1 (*exo*‐9‐hydroxymethylbicyclo [6.1.0] non‐4‐yne *N*‐succinimidyl carbonate) was synthesized from 1,5‐cyclooctadiene through sequential copper‐catalyzed cyclopropanation, epimerization, reduction, bromination, elimination and NHS functionalization (Scheme S1). PEG‐BCN was prepared from reacting 20 kDa 4‐arm PEG amine hydrochloride with 1 in the presence of *N*,*N*‐diisopropylethylamine in DMF. The degree of NHS functionalization of PEG‐BCN was determined to be *ca*. 95% (Figure S1) based on ^1^H NMR integrations. The highly modifiable skeleton of BPc allowed us to sequentially introduce two azido PEG chains for SPAAC and one sulfonyl fragment for water solubility. Carbamate intermediate 2 was made from 2,4‐dimethyl‐3‐ethylpyrrole through BODIPY core formation, hydrolysis, hydroxyl activation and carbamate formation. The second azido PEG chain was introduced via allylic bromination followed by nucleophilic substitution to afford compound 3, to which a sulfonyl side chain was attached following the same chemistry to give the final water‐soluble gel precursor BPcSO_3_. The photocleavable crosslinker, BPcSO_3_ is readily dissolvable in PBS at a concentration of 1 mM (Figure 1B). Compounds 1–3 and BPcSO_3_ were fully characterized by NMR spectroscopy (Figures S2–S14) and mass spectrometry.

### 2.2 Formation and Characterization of the Non‐Loaded Hydrogels

**FIGURE 2:**
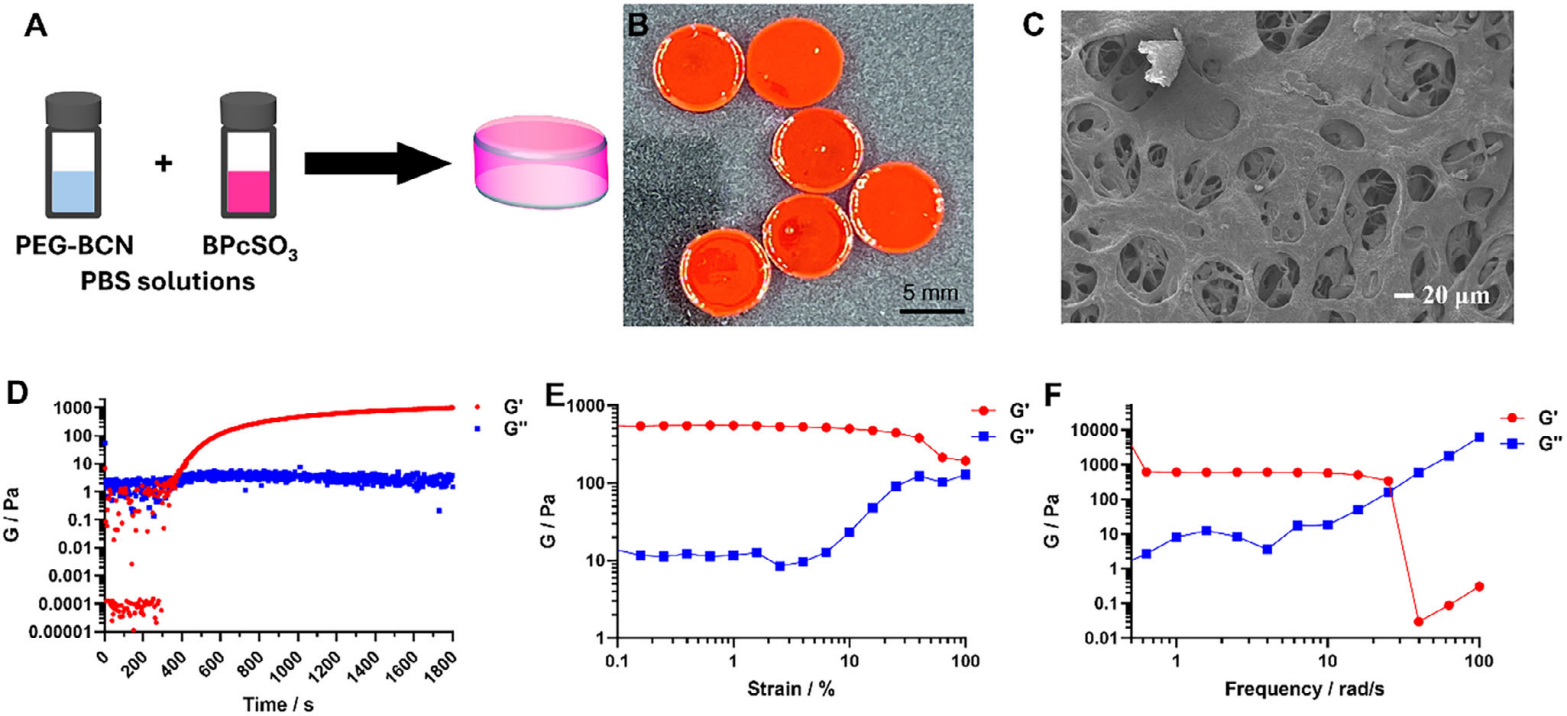
Formation and characterization of the non‐loaded hydrogels. (A) Gel preparation from mixing PBS solutions of **PEG**‐**BCN** and **BPcSO_3_**. (B) Image of the 4 wt% hydrogel discs demoulded from an 8‐millimeter PDMS mould. (C) SEM image of the 4 wt% hydrogel. (D) Gel properties of 4 wt% samples were monitored via time‐sweep experiments at fixed strain amplitude (1%) and frequency (5 rad/s) to measure G′ (red) and G″ (blue) of the hydrogels as a function of time at 298 K. (E) Gel properties of 4 wt% samples were monitored via strain‐sweep experiments at a fixed frequency (10 rad/s) to measure G′ (red) and G″ (blue) as a function of strain amplitude. (F) Gel properties of 4 wt% samples were monitored via frequency‐sweep experiments at a fixed strain (0.1%) to measure G′ (red) and G″ (blue) as a function of frequency.

We next screened gelation time and mechanical properties of the hydrogel formulations of different weight fractions (wt%) using rheology. Both gel precursors 4‐arm PEG‐BCN and 2‐arm BPcSO_3_ can be readily dissolved in PBS without the aid of organic solvents, and the hydrogel network can be formed via SPAAC (Figure 2A,B). No gelation was observed for 1 wt% pre‐gel mixtures (Figure S15) while we started to observe gelation at 2 wt% after 23.9 ± 2.1 min as indicated by a crossover point between the storage and loss modulus (G′ > G″) (Figure S16), yielding a soft gel with a storage modulus (G′) of 13 ± 2 Pa. Increasing the wt% of the formulation led to accelerated gelation kinetics and enhanced mechanical properties (Table 1 and Figure S17). The rheological results are consistent with the morphological observations from scanning electron microscopy (SEM) images of the non‐loaded gels, where the gel network becomes denser and more compact as the wt% of the gel increases (Figure 2C and Figure S18). At a concentration of 4 wt%, the hydrogels showed favorable biocompatibility in the absence/presence of light, with no significant differences in the cell viability of human dermal fibroblasts (Figure S19). Based on these results, we chose to work with a 4 wt% hydrogel formulation for our following studies as it gives a suitable gelation kinetics of 6.7 ± 0.7 min for injection (Figure 2D). Strain‐dependent oscillatory rheology (Figure 2E) of the 4 wt% hydrogel revealed a linear viscoelastic region up to 1% strain, with the material displaying a more fluid‐like behavior at higher strains. On the other hand, the frequency‐dependent rheological profile (Figure 2F) of the 4 wt% hydrogel suggested that the gel‐like property was maintained across the frequency range of 0.1–25 rad/s.

**TABLE 1:** Summary of the mechanical properties, gelation times and photodegradation half‐lives of the different hydrogel formulations.

Gel formulation	Storage modulus ^e^ (Pa)	Crossover timepoint ^g^ (min)	Photodegradation half‐life^h^ (min)
1 wt% ^a^	N/A	N/A	N/A
2 wt% ^a^	13 ± 2	23.9 ± 2.1	N/A
3 wt% ^a^	320 ± 11	11.9 ± 0.5	N/A
4 wt% ^a^	1032 ± 276	6.7 ± 0.7	5.1 ± 0.3; 2.4 ± 0.3 ^i^
4 wt% Control ^b^	744 ± 29 ^f^	28.2 ± 1.7	N/A
4 wt% + 100 nm PSNP (25%) ^c^	580 ± 27	9.0 ± 0.2	6.0 ± 0.5
4 wt% + OVA (25%) ^d^	593 ± 49	9.2 ± 0.4	7.9 ± 1.5

^a^ Gels of different wt% prepared from PBS solutions of **PEG**‐**BCN** (2 µM) and **BPcSO_3_** (90 µM) in a 1:2 molar ratio;

^b^ Gels (4 wt%) prepared by mixing the PBS solutions of **PEG**‐**BCN** (2 µM) and PEG‐Diazide (90 µM) in a 1:2 molar ratio;

^c^ Gels prepared by mixing the PBS solutions of **PEG**‐**BCN** (2 µM) and **BPcSO_3_** (90 µM) and aqueous suspension of PSNP‐COOH (50 mg/mL; 100 nm) in a 40:2:10 volume ratio;

^d^ Gels prepared by mixing the PBS solutions of **PEG**‐**BCN** (2 µM), **BPcSO_3_** (90 µM) and ovalbumin (40 mg/mL) in a 40:2:10 volume ratio;

^e^ G′ of the gels after 30 min of gelation;

^f^ G′ of the gels after complete gelation;

^g^ Time points when G′ exceeded G″ of the gels;

^h^ Photodegradation half‐life refers to the timepoint when the G′ of the gel drops down to 50% of the initial G′ of the gel (irradiation light intensity is 163 mW/cm^2^ unless otherwise noted);

^i^ Photodegradation half‐life determined under 555 mW/cm^2^ light irradiation. CoolLED light source (*λ* = 550 nm) was used for photodegradation studies herein. N/A: not applicable (N = 3).

### 2.3 Photodegradation Profile of the Non‐Loaded Hydrogels

**FIGURE 3:**
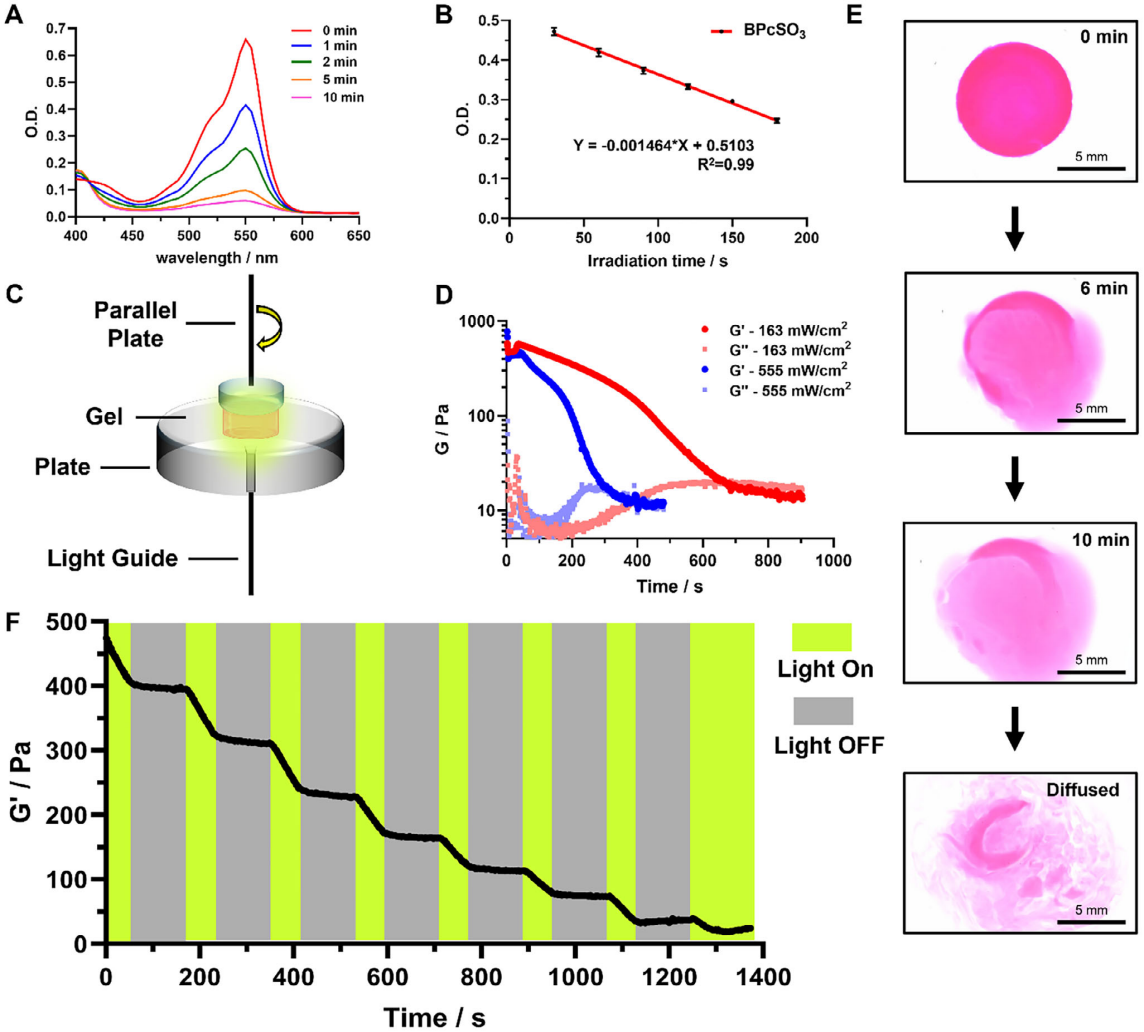
Light‐controlled degradation of the non‐loaded hydrogels. (A) UV/Vis spectra of **BPcSO_3_** (20 µM) in PBS at different timepoints of irradiation with a green LED array (*λ* = 530 nm; 15 mW/cm^2^). (B) Photo‐uncaging kinetics of **BPcSO_3_** (20 µM) in DMSO/H_2_O (1:10, *v*/*v*) under irradiation of a green LED array (*λ* = 530 nm; 15 mW/cm^2^). The plot is the absorbance of **BPcSO_3_** (*λ* = 550 nm) as a function of irradiation time. (C) Schematic of in situ rheological monitoring of the gel photodegradation. An 8‐millimeter hydrogel disc was loaded to the rheometer equipped with a transparent quartz plate, and light was introduced from the bottom through a light guide. (D) Light‐triggered gel degradation of 4 wt% samples under different intensities of light irradiation (*λ* = 550 nm). The plot shows changes in G′ of the gels as a function of irradiation time. (E) Images of an 8‐millimeter hydrogel disc after 0‐, 6‐ and 10‐minute irradiation (*λ* = 550 nm) at light intensity of 555 mW/cm^2^, and diffused gel residue after 10 min of irradiation. (F) Controlled photodegradation of the non‐loaded hydrogel. The hydrogel disc was illuminated with light for 1 min (*λ* = 550 nm; 163 mW/cm^2^), followed by 2‐minute irradiation pause. The illumination/irradiation pause cycle was continued until the gel was fully degraded (G′ falls below G″). The plot is the change in G′ of the gel as a function of operation time [55].

The photodegradation properties of the 4 wt% hydrogel formulation was then investigated. We first characterized the photocleavage process of BPcSO_3_ using UV/Vis spectroscopy. A PBS solution of BPcSO_3_ (20 µM) shows a strong absorption band at 550 nm (Figure 3A), with a molar absorption coefficient of 33,000 M^−1^·cm^−1^. Upon irradiation with a green LED array (*λ* = 530 nm; 15 mW/cm^2^), a gradual decrease in absorbance was observed as the irradiation time was increased with an isosbestic point observed at *λ* = 415 nm and complete photo‐uncaging achieved after 10 min of irradiation (Figure 3A). The photo‐uncaging quantum yield of BPcSO_3_ was determined to be 9.0 ± 0.3% (Figure 3B), which is an order of magnitude higher than the reference BODIPY photocage (Figure S20). The photocleavage reaction was also confirmed by LC‐MS analysis (Figure S21) [53]. We next studied the light‐triggered degradation kinetics of the non‐loaded hydrogels using an in situ irradiation set‐up in the rheometer (Figure 3C). As‐formed hydrogels were loaded to the quartz plate of the rheometer, and the moduli of the gels were recorded as a function of irradiation time. Hydrogel photodegradation was studied under irradiation at light intensities of 163 and 555 mW/cm^2^, respectively, and the corresponding photodegradation half‐lives were calculated to be 5.1 ± 0.3 and 2.4 ± 0.3 min (Figure 3D). Morphological changes of the hydrogel during the photodegradation can also be visualized: an 8‐millimeter pink‐orange colored hydrogel disc under light irradiation (555 mW/cm^2^) underwent a melting‐like process as the BPcSO_3_ linkers photo‐dissociated and finally transformed into a pink liquid (Figure 3E). To further validate the crucial role of BPcSO_3_ in photodegradation, we prepared control hydrogels using PEG‐BCN and PEG‐diazide linkers (M_n_ = 450 Da). The resulting gels exhibit similar mechanical properties but slower gel formation kinetics (28.2 ± 1.7 min; Figure S22). This slower gelation possibly results from the enhanced ionic complexation [54] and the slower diffusion of the less water‐soluble PEG linkers throughout the pre‐gel mixture and thus lower crosslinking efficiency. No signs of photodegradation were observed after 30 min of light irradiation (*λ* = 550 nm; Figure S25). These results indicate that the water‐soluble and photocleavable nature of BPcSO_3_ underlies the fast gelation and efficient photodegradation of the hydrogels. We next showed that the hydrogel degradation, where we alternated between irradiating the gel for one minute and withdrawing the light for another two minutes, can be controlled by light in a highly precise manner (Figure 3F), highlighting the advantage of using light as the external stimulus.

### 2.4 Encapsulation and Photo‐Release of Nanoparticles and Proteins in the Hydrogels

**FIGURE 4:**
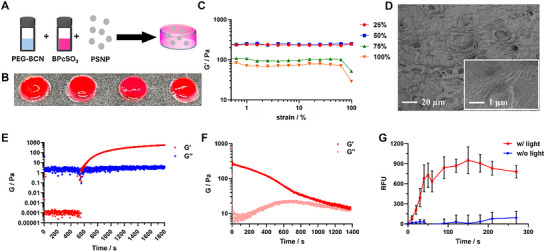
Encapsulation and photo‐release of nanoparticles in 4 wt% hydrogels. (A) Gel preparation from mixing PBS solutions of **PEG**‐**BCN** and **BPcSO_3_** and aqueous suspension of carboxylic PSNP (100 nm). (B) Image of the discs of different hydrogel‐PSNP composites demolded from an 8‐millimeter PDMS mold, representing the composites containing 25, 50, 75, and 100% of carboxylic PSNP, from left to right. (C) Gel properties of different hydrogel‐nanoparticle composites were monitored via strain‐sweep experiments at a fixed frequency (1 rad/s) to measure G′ as a function of strain amplitude. (D) SEM images of the hydrogel‐nanoparticle composite (25%). (E) Gel properties of the hydrogel‐nanoparticle composite (25%) were monitored via time‐sweep experiments at fixed strain amplitude (10%) and frequency (5 rad/s) to measure G′ (Red) and G″ (Blue) of the composites as a function of time at 298 K. (F). Light‐triggered degradation of the hydrogel‐nanoparticle composite (25%) under light irradiation (*λ* = 550 nm; 163 mW/cm^2^). The plot shows the change in G′ and G″ of the gel as a function of irradiation time. (G) Light‐controlled release of nanoparticles from the composites under irradiation of a green LED array (*λ* = 530 nm; 15 mW/cm^2^). Fluorescence intensity (*λ_em_* = 450 nm; RFU (relative fluorescence unit)) of blue carboxylic PSNP (100 nm) was monitored as a function of irradiation time. (N = 3).

Following the characterization of the SPAAC‐driven network formation and the photo‐uncaging‐mediated gel degradation, we began to explore the use of the photoresponsive hydrogel for nanoparticle loading and light‐controlled nanoparticle release. The mesh size of the gel network made from 4‐arm PEG‐BCN and 2‐arm BPcSO_3_ was estimated to be 64–83 nm (Supporting Information). Hence, we chose 100 nm carboxylic acid functionalized polystyrene nanoparticles (PSNP) as our model cargo. We first investigated hydrogel formation at different loading volume ratios (1:4, 2:4, 3:4, and 4:4) of a 50 mg/mL aqueous solution of PSNP to the 4 wt% pre‐gel PBS solution (Figure 4A and Table S1). Successful gelation was observed for all formulations (Figure 4B). The hydrogel‐nanoparticle composite containing 25% and 50% of the PSNP was found to exhibit larger G′ (Figure 4C). The increase in PSNP loading volume ratio led to a decrease in mechanical strength of the gels, where the composite containing 75% and 100% of the PSNP showed a decreased G′. The declining mechanical strength of the composites could result from spatial crowding imparted by the bulky PSNP that diminishes the degree of crosslinking. In strain‐dependent oscillatory rheology measurements (Figures S26–S33), 25% and 50% composites displayed a broad linear viscoelastic region without network failure up to 100% strain, while 75% and 100% composites started to fail at higher strains (> 60%). This result suggested that the incorporation of the rigid PSNP significantly enhanced the mechanical toughness of the gel network [56, 57], as the non‐loaded hydrogels failed at *ca*. 40% strain (Figure 2E). We next continued the characterization using the 25% hydrogel‐nanoparticle composite. SEM imaging of the gel containing 100 nm PSNP (25%, *v*/*v*) showcased a homogeneous and compact distribution of nanoparticles throughout the network (Figure 4D). Co‐gelation with the PSNP resulted in a slower crossover timepoint of 9.0 ± 0.2 min compared to the non‐loaded 4 wt% one (Figure 4E). On the other hand, the photodegradation half‐life of the 25% hydrogel‐nanoparticle composite was slightly increased to 6.0 ± 0.5 min (Figure 4F). We next loaded fluorescently labelled PSNP mixture (100 nm blue fluorescently labelled PSNP: non‐labelled PSNP = 2:8, *v*/*v*) to the 25% hydrogel‐nanoparticle composites to help monitor photo‐release of the nanoparticles in PBS during gel photodegradation. Under light irradiation, continuous release of the nanoparticles can be detected following the blue emission at 450 nm (*λ*_ex_ = 365 nm; Figure 4G). An increase in intensity (13.5 ± 5.9%) [58] was observed for the dark control over the course of incubation in PBS (Figure 4G), indicating that the hydrogel displayed a good encapsulation capacity for 100 nm PSNP. This result also showcases the potential of developing therapeutic hydrogel‐nanoparticle composites [59, 60], where the nanoparticles are loaded with therapeutics, for sustained and light‐controlled drug release.

We also studied the encapsulation and release of smaller protein cargo, i.e., ovalbumin which is *ca*. 8 nm in size and is widely used in preclinical vaccine development. Likewise, we first screened the gelation at different mixing volume ratios (1:4, 2:4, 3:4, and 4:4) of ovalbumin (40 mg/mL) in PBS to the 4 wt% pre‐gel PBS solution (Table S2 and Figures S34–S42). Efficient gelation was observed for most of the formulations, except for 100% loading, and a similar trend in mechanical properties was established. The gels with higher loading of ovalbumin exhibited a smaller G′ compared to the hydrogels with lower loading (Figure S42C). Slight decreases in gelation (9.2 ± 0.4 min) and photodegradation (7.9 ± 1.5 min) kinetics of ovalbumin‐loaded gels were observed compared to PSNP‐loaded formulations. Unlike the hydrogel‐nanoparticle composite, we observed protein aggregates in SEM images of the lyophilized gel composed of ovalbumin (25%, *v*/*v*) (Figures S42D and S43). Since the protein size (8 nm) is below the mesh size (64–83 nm) of the gel, we observed a substantial leakage of CF647 labelled ovalbumin in PBS as also evidenced by the attenuated color of the gel discs (Figure S42G) [61].

### 2.5 Ex Vivo Demonstrations of Injectability and Degradation

**FIGURE 5:**
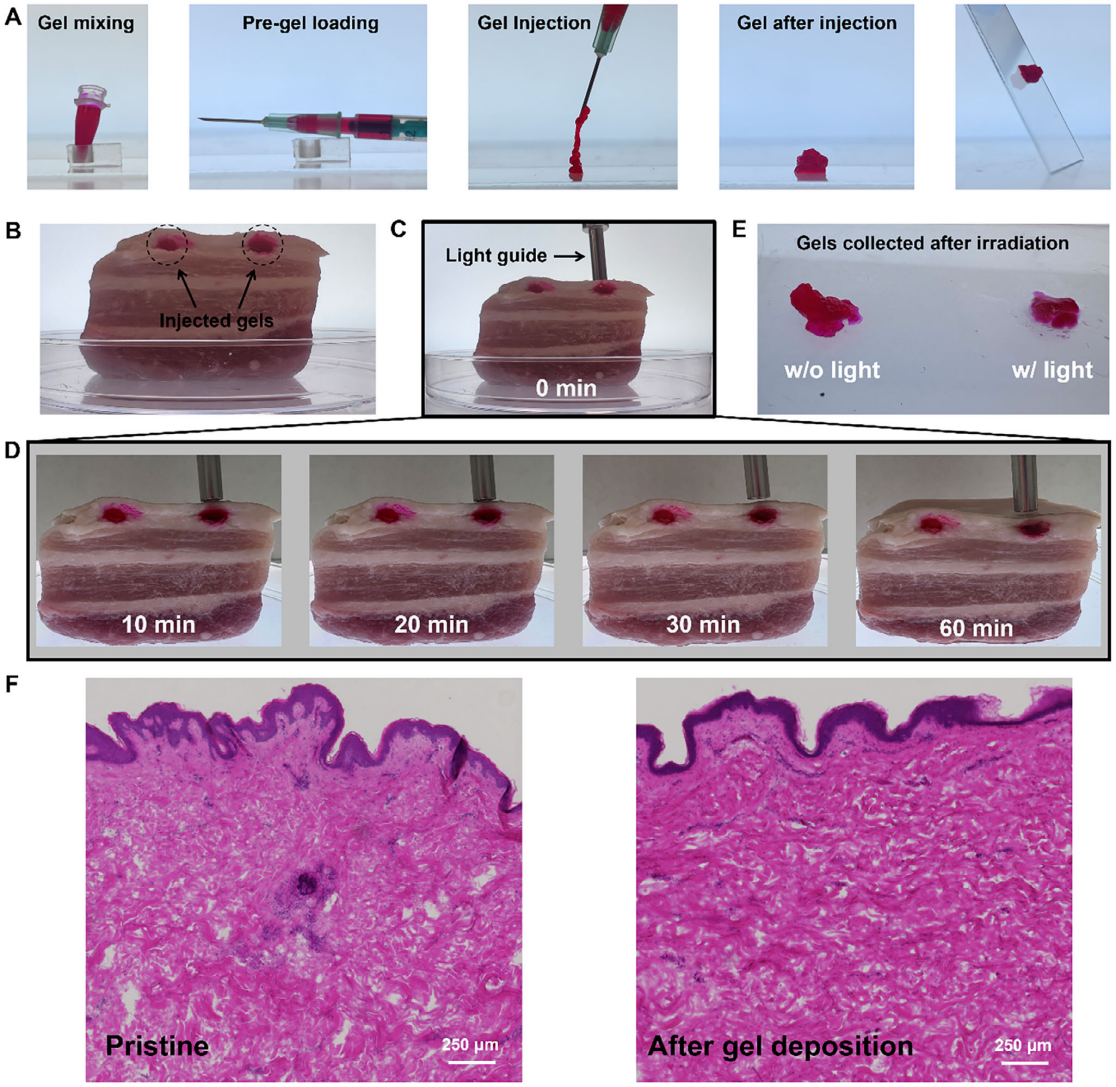
Ex vivo demonstration in a porcine model and gel biocompatibility testing in human skin. (A) Gel (200 µL) injection using a syringe. B) Image of a porcine belly (5 × 5 × 4 cm (L × W × H)) with two 100 µL gels deposited through injection. (C) Image of the gels in porcine belly before light irradiation. (D) Images of the gels in porcine belly after 10, 20, 30, and 60 min of light irradiation (*λ* = 550 nm; 163 mW/cm^2^). (E) Image of the gels retrieved from the porcine tissue after light irradiation. (F) Micrographs of hematoxylin and eosin‐stained human skin tissue seven days after intradermal gel injection (right) or no injection (left; scale bars = 250 µm).

We finally demonstrated the injectability of this hydrogel formulation and its potential for subcutaneous drug delivery in ex vivo porcine tissues. The gel mixture prior to complete gelation is very fluid (Figure 2D) and can be directly aspirated into a syringe through a 21G needle (Figure 5A). The pre‐gel can be easily injected out of the same needle after an extended period of gelation in the syringe (*ca*. 5 min), ensuring sufficient time for handling. The force required to inject the pre‐gel through a 21G or 23G needle is within a range of 1 to 3 Newtons (Figures S44 and S45). Following this procedure, we injected two 100 µL gels beneath the porcine skin (*ca*. 5 mm depth; Figure 5B). Next, we introduced light irradiation (*λ* = 550 nm) through a light guide positioned atop one of the gel deposits (Figure 5C). After 60 min of light treatment (555 mW/cm^2^; Figure 5D), the size of the irradiated gel was reduced by *ca*. 50% and became darker in color compared to the non‐irradiated counterpart (Figure 5E and Figure S46), indicative of effective gel photodegradation. Next, we evaluated the injection feasibility and biocompatibility of gel without light irradiation in donated human skin ex vivo. Healthy, clinically‐resected abdominal skin was biopsied to isolate explants composed of full thickness epidermis and dermis [62], injected intradermally, and cultured for one week. Intradermal injection of the pre‐gel solution met with no resistance and resulted in a visible pink bleb that gradually faded over the course of one week (Figure S47). The degradation of the gel is likely attributable to its softness and the superficial deposition, together with its hydrolysable chemical structure. Histological evaluation revealed no discernible trace of gel, no disturbance of the collagen fibril ultrastructure in the dermis, nor any signs of acute toxicity (Figure 5F). Thus, we conclude that the low wt% gels developed herein are promising candidates for short‐term light‐controlled release of superficially injected drug depots.

## 3 Conclusions

To conclude, we developed an injectable and photodegradable hydrogel formulation based on catalyst‐free click chemistry (i.e., SPAAC) which consists of water‐soluble azido BPcSO_3_ and BCN‐functionalized PEG‐BCN. Gelation takes place in minutes even at very low wt% and the formed gels undergo precise and light‐controlled degradation. The in situ gelation properties enable the formulation to be aspirated and injected prior to complete gelation. This hydrogel system can encapsulate nanoparticles and proteins upon gel formation, and can photo‐release the contents following the network degradation mediated by the photocleavage of BPc. PSNP cargo with a size over the mesh size of the hydrogel exhibits minimal diffusional release, while the smaller ovalbumin shows appreciable cargo leakage. We demonstrated the potential use of the hydrogel formulation for subcutaneous drug delivery in an ex vivo porcine model, where the pre‐gel hybrid mixture could be injected beneath the porcine skin and continue to gel in situ after which the light‐controlled degradation could be triggered by green light. Straightforward loading, injection, and degradation in human skin further highlight the promise of the developed hydrogel system for photo‐modulated therapeutic release in superficial tissues in a clinically relevant setting. We also envision the application of this formulation in many other exciting research areas encompassing mechanobiology [63, 64], organoids [65, 66, 67] and tissue engineering [68, 69, 70], among others [71, 72, 73].

## 4 Experimental Section

### 4.1 Synthesis

Detailed synthesis and characterization of 4‐arm PEG‐*exo*‐BCN and **BPcSO_3_** can be found in the supporting information.

### 4.2 Spectroscopy

UV–vis and fluorescence spectra were recorded on a Molecular Devices Spectramax M5 plate reader. 6‐well cell culture polystyrene plates (Corning Incorporated, 3516) and 96‐well quartz plates (Hellma Analytics, 73009‐B‐44) were used to load samples prepared.

### 4.3 Photoirradiation Setup

Irradiation experiments were conducted with 1) a Teleopto LAD‐1 light‐emitting diode (LED) array driver powering a LEDA‐530 LED array (*λ*  =  530 nm) purchased from Bio Research Center Co., Ltd. (Japan) and 2) a CoolLED pE‐300ultra Illumination System (including a Light Source, a Control Pod, a set of three Excitation Filter Holders (405, 450 and 550 nm; 25 mm dia.) and a Power Supply) coupled with a liquid light guide (pE‐1906) purchased from Scientific Alba Ltd. (UK). Light intensity was measured using a Thorlabs PM100D power meter equipped with a S121C photodiode.

### 4.4 Scanning Electron Microscopy

Hydrogels for scanning electron microscopy imaging were freeze‐dried and transferred to conductive tape. Samples were coated with 15 nm chromium in a pumped sputter coater (Quorum, Q150T Plus) before scanning electron imaging was conducted on a Zeiss Auriga microscope with 5 kV voltage, or they were coated with gold (30 s deposition time, 20 mA current) via sputtering deposition (Emitech K575X peltier cooled) before scanning electron imaging was conducted on a Zeiss Leo Gemini with 3 kV accelerating voltage.

### 4.5 Rheological and Injection Force Measurements

Rheological measurements were done using a rheometer (Anton‐Paar MCR 302) with a stainless‐steel measuring plate of 8 mm diameter and quartz plate with peltier control (P‐PTD 200/GL). Light source (CoolLED pE‐300ultra) was introduced to the bottom of the quartz plate through a liquid light guide. Injection forces were measured using a UniVert 1 kN mechanical test machine (CellScale, Waterloo Canada).

### 4.6 Cell Viability

Human dermal fibroblasts (HDF; Adult) were purchased from Merck (106‐05A). HDF were seeded into a 6‐well plate at a density of 100,000 cells per well and incubated overnight at 37°C with 5% CO_2_ overnight. The cells were then treated with either 4 wt% hydrogels, 4 wt% hydrogels combined with 30‐min light irradiation (*λ* = 530 nm; 15 mW/cm^2^), or 30‐min light irradiation alone (*λ* = 530 nm; 15 mW/cm^2^). After 48 h, 10% (v/v) alamarBlue reagent (DAL1100, Invitrogen, Waltham, MA, USA) was added to assess cell viability. Following a 2‐hour incubation, fluorescence (FL) intensity was recorded at Ex545/Em600 using a multimode microplate reader (SpectraMax M5, Molecular Devices, US). Cell viability was calculated using the following formula and expressed as mean ± SD: Cell Viability (%) = (FL intensity−FL intensity of reagent only)/(mean FL intensity of the control group−FL intensity of reagent only) × 100%.

### 4.7 Histology

Fresh human skin tissue was obtained from an elective abdominoplasty performed at Charing Cross Hospital, Imperial NHS Trust, London, UK. The patient signed an informed consent form describing procedures following Local Research Ethics Committee protocols. Human skin tissues were handled with an Imperial College Healthcare Tissue Bank‐approved license (17/WA/0161). Resected skin was further processed into ≈1 cm^3^ square biopsies including the full thickness epidermis and dermis with most subcutaneous fat removed. Injections volumes of 50 µL were performed at a 15 degree angle at 1 mm depth, with a non‐injected tissue incubated in identical conditions to control for changes in tissue volume and viability. Explants were injected intradermally with a 50 µL of pre‐gel solution using a 23 G needle and cultured in 12‐well plates with 2 mL of complete DMEM (Gibco) for one week, with cell culture media replaced daily. After seven days, both gel‐injected and control (non‐injected) skin explants (N = 3 each) were washed with PBS and immersed in PBS + 4% paraformaldehyde at 4°C for 36 h, followed by incubation in PBS + 30% sucrose (w/v) at 4°C for 36 h, followed by embedding in Optimal Cutting Temperature (Tissue‐Tek) compound for cryosectioning. Cryosections were obtained at 10 µm‐thickness and processed at the Francis Crick Institute Histology Core.

### 4.8 Statistical Analysis

All data analyses were performed using Prism software (GraphPad) and presented as the mean ± SD based on three independent experiments unless otherwise noted.

## Author Contributions

B.S. conceived and led the project under the supervision of M.M.S. B.S. synthesized and characterized the gel precursors, studied the gelation and photodegradation, investigated the cargo loading and photo‐release, and carried out the ex vivo experiments in porcine tissues. D.J.P. provided functionalized ovalbumin and did the ex vivo experiments in human skin. T.F.D. and K.Z. collected the SEM images. Y.C. tested the cell viability of the hydrogels. Y.S. conducted injection force measurements. J.P.W. and K.Z. contributed to the hydrogel preparation, rheological measurements and data acquisition. R.J.S. contributed skin explant access and cultures. B.S. drafted the paper with input from all authors. All the authors helped in evaluating the results and commented on the manuscript.

## Conflicts of Interest

M.M.S. invested in, consults for or was on scientific advisory boards or boards of directors, and conducts sponsored research funded by companies related to the biomaterials field. The rest of the authors declare no conflicts of interest.

## Supporting information




**Supporting File**: smll74162‐sup‐0001‐SuppMat.docx.

## Data Availability

The data that support the findings of this study are available from the corresponding author upon reasonable request.

## References

[1] K. Hüll , J. Morstein , and D. Trauner , “In Vivo Photopharmacology,” Chemical Review 118 (2018): 10710. 10.1021/acs.chemrev.8b00037 29985590

[2] P. Kobauri , F. J. Dekker , W. Szymanski , and B. L. Feringa , “Rational Design in Photopharmacology With Molecular Photoswitches,” Angewandte Chemie International Edition 62 (2023): 202300681, 10.1002/anie.202300681. 37026576

[3] D. Hartmann , J. M. Smith , G. Mazzotti , R. Chowdhry , and M. J. Booth , “Controlling Gene Expression With Light: A Multidisciplinary Endeavour,” Biochemical Society Transactions 48 (2020): 1645–1659, 10.1042/BST20200014. 32657338 PMC7458398

[4] A. M. Kloxin , A. M. Kasko , C. N. Salinas , and K. S. Anseth , “Photodegradable Hydrogels for Dynamic Tuning of Physical and Chemical Properties,” Science 324 (2009): 59–63, 10.1126/science.1169494. 19342581 PMC2756032

[5] N. Gjorevski , M. Nikolaev , T. E. Brown , et al., “Tissue Geometry Drives Deterministic Organoid Patterning,” Science 375 (2022): 6576, 10.1126/science.aaw9021. PMC9131435 34990240

[6] M. Karimi , P. S. Zangabad , S. Baghaee‐Ravari , M. Ghazadeh , H. Mirshekari , and M. R. Hamblin , “Smart Nanostructures for Cargo Delivery: Uncaging and Activating by Light,” Journal of the American Chemical Society 139 (2017): 4584–4610, 10.1021/jacs.6b08313. 28192672 PMC5475407

[7] X. Li , J. F. Lovell , J. Yoon , and X. Chen , “Clinical Development and Potential of Photothermal and Photodynamic Therapies for Cancer,” Nature Reviews Clinical Oncology 17 (2020): 657–674, 10.1038/s41571-020-0410-2. 32699309

[8] Z. Zhang , W. Wang , M. O'Hagan , J. Dai , J. Zhang , and H. Tian , “Stepping Out of the Blue: From Visible to Near‐IR Triggered Photoswitches,” Angewandte Chemie International Edition 61 (2022): 202205758, 10.1002/anie.202205758. 35524420

[9] S. M. Won , E. Song , J. T. Reeder , and J. A. Rogers , “Emerging Modalities and Implantable Technologies for Neuromodulation,” Cell 181 (2020): 115–135, 10.1016/j.cell.2020.02.054. 32220309

[10] A. Bansal , S. Shikha , and Y. Zhang , “Towards Translational Optogenetics,” Nature Biomedical Engineering 7 (2023): 349–369, 10.1038/s41551-021-00829-3. 35027688

[11] R. Raman , T. Hua , D. Gwynne , et al., “Light‐degradable Hydrogels as Dynamic Triggers for Gastrointestinal Applications,” Science Advances 6 (2020): aay0065, 10.1126/sciadv.aay0065. PMC6968934 32010768

[12] A. H. C. Anthis , S. Kilchenmann , M. Murdeu , et al., “Reversible Mechanical Contraception and Endometriosis Treatment Using Stimuli‐Responsive Hydrogels,” Advanced Materials 36 (2024): 2310301, 10.1002/adma.202310301. 38298130

[13] L. Tomasini , M. Ferrere , and J. Nicolas , “Subcutaneous Drug Delivery From Nanoscale Systems,” Nature Reviews Bioengineering 2 (2024): 501–520, 10.1038/s44222-024-00161-w.

[14] I. Usach , R. Martinez , T. Festini , and J.‐E. Peris , “Subcutaneous Injection of Drugs: Literature Review of Factors Influencing Pain Sensation at the Injection Site,” Advances in Therapy 36 (2019): 2986–2996, 10.1007/s12325-019-01101-6. 31587143 PMC6822791

[15] C. Ash , M. Dubec , K. Donne , and T. Bashford , “Effect of Wavelength and Beam Width on Penetration in Light‐tissue Interaction Using Computational Methods,” Lasers in Medical Science 32 (2017): 1909–1918, 10.1007/s10103-017-2317-4. 28900751 PMC5653719

[16] L. Finlayson , I. R. M. Barnard , L. McMillan , et al., “Depth Penetration of Light Into Skin as a Function of Wavelength From 200 to 1000 Nm,” Photochemistry and Photobiology 98 (2022): 974–981, 10.1111/php.13550. 34699624

[17] N. A. Peppas , J. Z. Hilt , A. Khademhosseini , and R. Langer , “Hydrogels in Biology and medicine: From Molecular Principles to Bionanotechnology,” Advanced Materials 18 (2006): 1345.

[18] J. Li and D. J. Mooney , “Designing Hydrogels for Controlled Drug Delivery,” Nature Reviews Materials 1 (2016): 16071, 10.1038/natrevmats.2016.71. PMC5898614 29657852

[19] K. Sano , Y. Ishida , and T. Aida , “Synthesis of Anisotropic Hydrogels and Their Applications,” Angewandte Chemie International Edition 57 (2018): 2532–2543, 10.1002/anie.201708196. 29034553

[20] S. Correa , A. K. Grosskopf , H. L. Hernandez , et al., “Translational Applications of Hydrogels,” Chemical Reviews 121 (2021): 11385–11457, 10.1021/acs.chemrev.0c01177. 33938724 PMC8461619

[21] H. Cao , L. Duan , Y. Zhang , J. Cao , and K. Zhang , “Current Hydrogel Advances in Physicochemical and Biological Response‐driven Biomedical Application Diversity,” Signal Transduction and Targeted Therapy 2021, 6, 426. 34916490 10.1038/s41392-021-00830-x PMC8674418

[22] K. Cui and J. P. Gong , “Aggregated Structures and Their Functionalities in hydrogels,” Aggregate 2 (2021): 33.

[23] R. Zhong , S. Talebian , B. B. Mendes , et al., “Hydrogels for RNA Delivery,” Nature Materials 22 (2023): 818–831, 10.1038/s41563-023-01472-w. 36941391 PMC10330049

[24] P. Lu , D. Ruan , M. Huang , et al., “Harnessing the Potential of Hydrogels for Advanced Therapeutic Applications: Current Achievements and Future Directions,” Signal Transduction and Targeted Therapy 9 (2024): 166, 10.1038/s41392-024-01852-x. 38945949 PMC11214942

[25] M. Liu , X. Zeng , C. Ma , et al., “Injectable Hydrogels for Cartilage and Bone Tissue Engineering,” Bone Research 5 (2017): 17014, 10.1038/boneres.2017.14. 28584674 PMC5448314

[26] F. Rizzo and N. S. Kehr , “Recent Advances in Injectable Hydrogels for Controlled and Local Drug Delivery,” Advanced Healthcare Materials 10 (2021): 2001341, 10.1002/adhm.202001341. 33073515

[27] M. Nguyen , M. Karkanitsa , and K. L. Christman , “Design and Translation of Injectable Biomaterials,” Nature Reviews Bioengineering 2 (2024): 810–828, 10.1038/s44222-024-00213-1.

[28] A. Nishiguchi , “Advances in Injectable Hydrogels With Biological and Physicochemical Functions for Cell Delivery,” Polymer Journal 56 (2024): 895–903, 10.1038/s41428-024-00934-5.

[29] A. P. Gorka , R. R. Nani , J. Zhu , S. Mackem , and M. J. Schnermann , “A near‐IR Uncaging Strategy Based on Cyanine Photochemistry,” Journal of the American Chemical Society 136 (2014): 14153–14159, 10.1021/ja5065203. 25211609 PMC4195383

[30] P. P. Goswami , A. Syed , C. L. Beck , et al., “BODIPY‐derived Photoremovable Protecting Groups Unmasked With Green Light,” Journal of the American Chemical Society 137 (2015): 3783–3786, 10.1021/jacs.5b01297. 25751156

[31] A. R. Sekhar , Y. Chitose , J. Janoš , et al., “Porphyrin as a Versatile Visible‐light‐activatable Organic/Metal Hybrid Photoremovable Protecting Group,” Nature Communications 13 (2022): 3614, 10.1038/s41467-022-31288-2. PMC9232598 35750661

[32] G. Alachouzos , A. M. Schulte , A. Mondal , W. Szymanski , and B. L. Feringa , “Computational Design, Synthesis, and Photochemistry of Cy7‐PPG, an Efficient NIR‐Activated Photolabile Protecting Group for Therapeutic Applications**,” Angewandte Chemie International Edition 61 (2022): 202201308, 10.1002/anie.202201308. PMC9311213 35181979

[33] A. M. Schulte , G. Alachouzos , W. Szymański , and B. L. Feringa , “Strategy for Engineering High Photolysis Efficiency of Photocleavable Protecting Groups Through Cation Stabilization,” Journal of the American Chemical Society 144 (2022): 12421–12430, 10.1021/jacs.2c04262. 35775744 PMC9284546

[34] A. Egyed , K. Németh , T. Á. Molnár , M. Kállay , P. Kele , and M. Bojtár , “Turning Red Without Feeling Embarrassed─ Xanthenium‐based Photocages for Red‐light‐activated Phototherapeutics,” Journal of the American Chemical Society 145 (2023): 4026–4034, 10.1021/jacs.2c11499. 36752773 PMC9951246

[35] T. L. Rapp and C. A. DeForest , “Tricolor Visible Wavelength‐selective Photodegradable Hydrogel Biomaterials,” Nature Communications 14 (2023): 5250, 10.1038/s41467-023-40805-w. PMC10462736 37640707

[36] M. Russo , H. Janeková , D. Meier , M. Generali , and P. Štacko , “Light in a Heartbeat: Bond Scission by a Single Photon Above 800 Nm,” Journal of the American Chemical Society 146 (2024): 8417–8424, 10.1021/jacs.3c14197. 38499198 PMC10979397

[37] D. R. Griffin and A. M. Kasko , “Photodegradable Macromers and Hydrogels for Live Cell Encapsulation and Release,” Journal of the American Chemical Society 134 (2012): 13103–13107, 10.1021/ja305280w. 22765384 PMC4180708

[38] J. M. Silva , E. Silva , and R. L. Reis , “Light‐triggered Release of Photocaged Therapeutics‐Where Are We Now?,” Journal of Controlled Release 298 (2019): 154–176, 10.1016/j.jconrel.2019.02.006. 30742854

[39] L. Li , J. M. Scheiger , and P. A. Levkin , “Design and Applications of Photoresponsive Hydrogels,” Advanced Materials 31 (2019): 1807333, 10.1002/adma.201807333. 30848524 PMC9285504

[40] B. M. Vickerman , E. M. Zywot , T. K. Tarrant , and D. S. Lawrence , “Taking Phototherapeutics From Concept to Clinical Launch,” Nature Reviews Chemistry 5 (2021): 816–834, 10.1038/s41570-021-00326-w. 37117665 PMC8493544

[41] P. J. LeValley , B. P. Sutherland , J. Jaje , et al., “On‐Demand and Tunable Dual Wavelength Release of Antibodies Using Light‐Responsive Hydrogels,” ACS Applied Bio Materials 3 (2020): 6944–6958, 10.1021/acsabm.0c00823. PMC8315695 34327309

[42] P. J. LeValley , R. Neelarapu , B. P. Sutherland , S. Dasgupta , C. J. Kloxin , and A. M. Kloxin , “Photolabile Linkers: Exploiting Labile Bond Chemistry to Control Mode and Rate of Hydrogel Degradation and Protein Release,” Journal of the American Chemical Society 142 (2020): 4671–4679, 10.1021/jacs.9b11564. 32037819 PMC7267699

[43] Y. Yang , K. Long , Y. Chu , H. Lu , W. Wang , and C. Zhan , “Photoresponsive Drug Delivery Systems: Challenges and Progress,” Advanced Functional Materials 34 (2024): 2402975, 10.1002/adfm.202402975.

[44] I. Kopyeva , R. P. Brady , and C. A. DeForest , “Light‐based Fabrication and 4D Customization of Hydrogel Biomaterials,” Nature Reviews Bioengineering 3 (2025): 159–180, 10.1038/s44222-024-00234-w. PMC12338255 40800012

[45] I. Batalov , J. R. Filteau , R. M. Francis , et al., “Grayscale 4D Biomaterial Customization at High Resolution and Scale,” bioRxiv (2024), 10.1101/2024.01.31.578280.

[46] X. Wang , C. Wang , Q. Zhang , and Y. Cheng , “Near Infrared Light‐responsive and Injectable Supramolecular Hydrogels for on‐demand Drug Delivery,” Chemical Communications 52 (2016): 978–981, 10.1039/C5CC08391E. 26588349

[47] X. Wang , C. Wang , X. Wang , Y. Wang , Q. Zhang , and Y. Cheng , “A Polydopamine Nanoparticle‐knotted Poly (ethylene glycol) Hydrogel for on‐demand Drug Delivery and Chemo‐photothermal Therapy,” Chemistry of Materials 29 (2017): 1370–1376, 10.1021/acs.chemmater.6b05192.

[48] C. Ruan , C. Liu , H. Hu , et al., “NIR‐II Light‐modulated Thermosensitive Hydrogel for Light‐triggered Cisplatin Release and Repeatable Chemo‐photothermal Therapy,” Chemical Science 10 (2019): 4699–4706, 10.1039/C9SC00375D. 31123581 PMC6496981

[49] N. E. Gregorio , F. Zhang , Y. Suita , et al., “PhoCoil: A Photodegradable and Injectable Single‐component Recombinant Protein Hydrogel for Minimally Invasive Delivery and Degradation,” Science Advances 11 (2025): adx3472, 10.1126/sciadv.adx3472. PMC12383250 40864698

[50] P. Shrestha , D. Kand , R. Weinstain , and A. H. Winter , “meso‐Methyl BODIPY Photocages: Mechanisms, Photochemical Properties, and Applications,” Journal of the American Chemical Society 145 (2023): 17497–17514, 10.1021/jacs.3c01682. 37535757

[51] C. A. DeForest , B. D. Polizzotti , and K. S. Anseth , “Sequential Click Reactions for Synthesizing and Patterning Three‐dimensional Cell Microenvironments,” Nature Materials 8 (2009): 659–664, 10.1038/nmat2473. 19543279 PMC2715445

[52] E. R. Ruskowitz and C. A. DeForest , “Photoresponsive Biomaterials for Targeted Drug Delivery and 4D Cell Culture,” Nature Reviews Materials 3 (2018): 17087, 10.1038/natrevmats.2017.87.

[53] Meso‐CH_2_OH BODIPY was not observed. Me‐BPcSO_3_ was proposed as the major, identifiable photoproduct.

[54] C. Maltesh and P. Somasundaran , “Effect of Binding of Cations to Polyethylene Glycol on Its Interactions With Sodium Dodecyl Sulfate,” Langmuir 8 (1992): 1926–1930, 10.1021/la00044a008.

[55] In the first ca. 2 min of photoirradiation, G′ and G′′ of the hydrogels underwent changes that deviated from the trend (as observed in Figure 3D) possibly resulting from the enhanced contact between the parallel plate and the gels as the latter became more liquid‐like.

[56] S. Rose , A. Prevoteau , P. Elzière , D. Hourdet , A. Marcellan , and L. Leibler , “Nanoparticle Solutions as Adhesives for Gels and Biological Tissues,” Nature 505 (2014): 382–385, 10.1038/nature12806. 24336207

[57] E. A. Appel , M. W. Tibbitt , M. J. Webber , B. A. Mattix , O. Veiseh , and R. Langer , “Self‐assembled Hydrogels Utilizing Polymer–nanoparticle Interactions,” Nature Communications 6 (2015): 6295, 10.1038/ncomms7295. PMC4651845 25695516

[58] The change in intensity was determined by calculating the area under curve for the w/light and w/o light curves in Figure 4G.

[59] M. Hamidi , A. Azadi , and P. Rafiei , “Hydrogel Nanoparticles in Drug Delivery,” Advanced Drug Delivery Reviews 60 (2008): 1638–1649, 10.1016/j.addr.2008.08.002. 18840488

[60] Y. Jiang , N. Krishnan , J. Heo , R. H. Fang , and L. Zhang , “Nanoparticle–hydrogel superstructures for biomedical applications,” Journal of Controlled Release 324 (2020): 505–521, 10.1016/j.jconrel.2020.05.041. 32464152 PMC7429280

[61] The decrease in the cumulative release of CF647 indicated by the declining absorbance with light irradiation could result from photo‐oxidation of the CF647 dyes.

[62] B. D. Barbieri , D. J. Peeler , K. Samnuan , et al., “The Role of Helper Lipids in Optimising Nanoparticle Formulations of Self‐amplifying RNA,” The Journal of Controlled Release 374 (2024): 280. 39142355 10.1016/j.jconrel.2024.08.016

[63] U. Blache , E. M. Ford , B. Ha , et al., “Engineered Hydrogels for Mechanobiology,” Nature Reviews Methods Primers 2 (2022): 98, 10.1038/s43586-022-00179-7. PMC7614763 37461429

[64] S. J. P. Callens , D. Fan , I. A. J. van Hengel , et al., “Emergent Collective Organization of Bone Cells in Complex Curvature Fields,” Nature Communications 14 (2023): 855, 10.1038/s41467-023-36436-w. PMC9984480 36869036

[65] M. T. Kozlowski , C. J. Crook , and H. T. Ku , “Towards Organoid Culture Without Matrigel,” Communications Biology 4 (2021): 1387, 10.1038/s42003-021-02910-8. 34893703 PMC8664924

[66] Z. Zhao , X. Chen , A. M. Dowbaj , et al., “Organoids,” Nature Reviews Methods Primers 2 (2022): 94, 10.1038/s43586-022-00174-y. PMC10270325 37325195

[67] Z. Gan , X. Qin , H. Liu , J. Liu , and J. Qin , “Recent Advances in Defined Hydrogels in Organoid Research,” Bioactive Materials 28 (2023): 386. 37334069 10.1016/j.bioactmat.2023.06.004 PMC10273284

[68] K. Y. Lee and D. J. Mooney , “Hydrogels for Tissue Engineering,” Chemical Reviews 101 (2001): 1869–1880, 10.1021/cr000108x. 11710233

[69] M. M. Stevens and J. H. George , “Exploring and Engineering the Cell Surface Interface,” Science 310 (2005): 1135–1138, 10.1126/science.1106587. 16293749

[70] A. C. Daly , L. Riley , T. Segura , and J. A. Burdick , “Hydrogel Microparticles for Biomedical Applications,” Nature Reviews Materials 5 (2020): 20–43, 10.1038/s41578-019-0148-6. PMC8191408 34123409

[71] S. R. Caliari and J. A. Burdick , “A Practical Guide to Hydrogels for Cell Culture,” Nature Methods 13 (2016): 405–414, 10.1038/nmeth.3839. 27123816 PMC5800304

[72] J. Lou and D. J. Mooney , “Chemical Strategies to Engineer Hydrogels for Cell Culture,” Nature Reviews Chemistry 6 (2022): 726–744, 10.1038/s41570-022-00420-7. 37117490

[73] A. P. Dhand , M. D. Davidson , and J. A. Burdick , “Lithography‐based 3D printing of hydrogels,” Nature Reviews Bioengineering 3 (2025): 108–125, 10.1038/s44222-024-00251-9. PMC12269901 40678688

