# Supplementary material for: BODIPY Photocage‐Based Injectable Hydrogel for Light‐Controlled Nanoparticle Release
Source: Small. 2026 Jul 1;22(41):e74162. doi: 10.1002/smll.74162 (PMC13392740; doi:10.1002/smll.74162)
Supplement: Supplementary file 1 — Supporting File: smll74162‐sup‐0001‐SuppMat.docx. [file SMLL-22-e74162-s001.docx]

Supporting Information

BODIPY Photocage-based Injectable Hydrogel for Light-controlled Nanoparticle Release

Baihao Shao, David J. Peeler, Thomas F. F. Fernandez Debets, Jonathan P. Wojciechowski, Yue Shao, Yuxi Cheng, Kun Zhou, Robin J. Shattock and Molly M. Stevens*

B. Shao, D. J. Peeler, T. F. F. Fernandez Debets, J. P. Wojciechowski, Y. Shao, Y. Cheng and M. M. Stevens

Department of Physiology, Anatomy and Genetics, Department of Engineering Science and Kavli Institute for Nanoscience Discovery, University of Oxford, Oxford OX1 3QU, UK

E-mail: [molly.stevens@dpag.ox.ac.uk](mailto:molly.stevens@dpag.ox.ac.uk)

D. J. Peeler and R. J. Shattock

Department of Infectious Diseases, Imperial College London, London W2 1PG, UK

B. Shao, D. J. Peeler, Y. Cheng, K. Zhou and M. M. Stevens

Department of Materials, Department of Bioengineering, Institute of Biomedical Engineering, Imperial College London, London SW7 2AZ, UK

**Table of Contents**

1. **General Methods S3**
2. **Synthesis S6**
3. **NMR Characterization S10**
4. **Characterization of the non-loaded hydrogels S17**
5. **Photo-uncaging quantum yield S20**
6. **Characterization of the control hydrogels S22**
7. **Characterization of the PNSP-loaded hydrogels S24**
8. **Characterization of the ovalbumin-loaded hydrogels S29**
9. **Injection force measurement S36**
10. ***Ex vivo* demonstration in human skin explants S37**
11. **References S38**

**1 General Methods**

*General methods and materials*: All reagents and starting materials were purchased from commercial vendors and used as supplied unless otherwise indicated. All experiments were conducted under air unless otherwise noted. Compounds were purified by column chromatography using silica gel (40-63 μm; VWR chemicals) as stationary phase and solvents mixtures used during chromatography are reported as volume ratios unless otherwise noted. ^1^H NMR and ^13^C NMR spectra were recorded on a JEOL 400 NMR spectrometer, with working frequencies of 400 MHz for ^1^H nuclei, and 101 MHz for ^13^C nuclei, respectively. High resolution ESI mass spectra were obtained from a flow injection analysis performed on an ACQUITY I-Class PLUS UPLC System (Waters, Milford, MA, USA) coupled to an ACQUITY RDa mass spectrometer (Waters, Milford, MA, USA) equipped with an ESI probe, in positive or negative ion mode. Melting points were measured on a Cole-Parmer® MP80 series Stuart automatic digital melting point apparatus.

20 kDa 4-arm PEG amine hydrochloride was purchased from JenKem Technology (USA). A 0.1 g/mL THF solution of PEG-diazide was purchased from Sigma-Aldrich (913650). Polystyrene particles (50 mg/mL; 100 nm-COOH; ABPS-0010-C) and blue fluorescent particles (10 mg/mL; 100 nm-COOH; AFB-0010-COOH) were purchased from Abvigen (New Jersey, USA).

As in previous work,^S^^[[1]](#endnote-2)^ ovalbumin from chicken egg white (OVA; Sigma Aldrich) was dissolved at 40 mg/mL in 0.1 M sodium bicarbonate (213.5 mg; 5 µmol) and placed under magnetic stirring at room temperature. An equivalent molar quantity of CF™ 647 succinimidyl ester (CF647; Sigma Aldrich) was dissolved at 10 mM in anhydrous DMSO, added dropwise into the stirred OVA solution, and allowed to react for 2 hours. The CF647-OVA product was purified by dialysis (Snakeskin 10 kDa cutoff; Sigma Aldrich) against water and lyophilized for future use.

Boneless porcine bellies were purchased from Medmeat Ltd. (Rochdale, UK), and cut into *ca.* 5🞨5🞨4 cm (L🞨W🞨H) sized tissue blocks for the experiments.

LC-MS chromatograms and ESI-MS spectra were measured on a Shimadzu LCMS-2050. Samples were measured using a Phenomenex Kinetex^®^ Evo C18 column (50 × 2.1 mm, 2.6 µm, 100 Å) at a flow rate = 1 mL/min, injection volume = 5 µL, detection wavelengths = 190 nm and 254 nm, and column temperature = 313 K. Mobile phases were: A = HPLC grade water with 0.1% formic acid (v/v) and B = HPLC grade acetonitrile with 0.1% formic acid (v/v). Samples were separated using a gradient elution from 5-95% B over 8 or 12 min.

*Spectroscopy*: UV-Vis and fluorescence spectra were recorded on a Molecular Devices Spectramax M5 plate reader. 6-well cell culture polystyrene plates (Corning Incorporated, 3516) and 96-well quartz plates (Hellma Analytics, 73009-B-44) were used to load samples prepared.

*Photoirradiation setup*: Irradiation experiments were conducted with 1) a Teleopto LAD-1 light-emitting diode (LED) array driver powering a LEDA-530 LED array (*λ* = 530 nm) purchased from Bio Research Center Co., Ltd. (Japan) and 2) a CoolLED pE-300ultra Illumination System (including a Light Source, a Control Pod, a set of three Excitation Filter Holders (405, 450 and 550 nm; 25 mm dia.) and a Power Supply) coupled with a liquid light guide (pE-1906) purchased from Scientific Alba Ltd. (UK). Light intensity was measured using a Thorlabs PM100D power meter equipped with a S121C photodiode.

*Scanning electron microscopy*: Hydrogels for scanning electron microscopy (SEM) imaging were freeze-dried and transferred to conductive tape. Samples were coated with 15 nm chromium in a pumped sputter coater (Quorum, Q150T Plus) before scanning electron imaging was conducted on a Zeiss Auriga microscope with 5 kV voltage, or they were coated with gold (30 s deposition time, 20 mA current) *via* sputtering deposition (Emitech K575X peltier cooled) before scanning electron imaging was conducted on a Zeiss Leo Gemini with 3 kV accelerating voltage.

*Rheological and injection force measurements*: Rheological measurements were done using a rheometer (Anton-Paar MCR 302) with a stainless-steel measuring plate of 8 mm diameter and quartz plate with peltier control (P-PTD 200/GL). Light source (CoolLED pE-300ultra) was introduced to the bottom of the quartz plate through a liquid light guide. Injection forces were measured using a UniVert 1 kN mechanical test machine (CellScale, Waterloo Canada).

*Cell viability*: Human dermal fibroblasts were seeded into a 6-well plate at a density of 100,000 cells per well and incubated overnight at 37°C with 5% CO₂ overnight. The cells were then treated with either 4 wt% hydrogels, 4 wt% hydrogels combined with 30-min light irradiation (*λ* = 530 nm; 15 mW/cm²), or 30-min light irradiation alone (*λ* = 530 nm; 15 mW/cm²). After 48 hours, 10% (v/v) alamarBlue reagent (DAL1100, Invitrogen, Waltham, MA, USA) was added to assess cell viability. Following a 2-hour incubation, fluorescence (FL) intensity was recorded at Ex545/Em600 using a multimode microplate reader (SpectraMax M5, Molecular Devices, US). Cell viability was calculated using the following formula and expressed as mean ± SD: Cell Viability (%) = (FL intensity - FL intensity of reagent only)/(mean FL intensity of the control group - FL intensity of reagent only)×100 %. Mean FL intensity of the control group refers to an average of FL intensity of the control group (n = 3).

*Histology*: Fresh human skin tissue was obtained from an elective abdominoplasty performed at Charing Cross Hospital, Imperial NHS Trust, London, UK. The patient signed an informed consent form describing procedures following Local Research Ethics Committee protocols. Resected skin was further processed into ~1 cm^3^ square biopsies including the full thickness epidermis and dermis with most subcutaneous fat removed. Injections volumes of 50 µL were performed at a 15 degree angle at 1 mm depth, with a non-injected tissue incubated in identical conditions to control for changes in tissue volume and viability. Explants were injected intradermally with a 50 µL of pre-gel solution using a 23 G needle and cultured in 12-well plates with 2 mL of complete DMEM (Gibco) for one week, with cell culture media replaced daily. After seven days, both gel-injected and control (non-injected) skin explants (N = 3 each) were washed with PBS and immersed in PBS + 4% paraformaldehyde at 4 °C for 36 hours, followed by incubation in PBS +30% (w/v) sucrose at 4 °C for 36 hours, followed by embedding in Optimal Cutting Temperature (Tissue-Tek) compound for cryosectioning. Cryosections were obtained at 10 µm-thickness and processed at the Francis Crick Institute Histology Core.

*Mesh size estimation*: The arm length of 20k 4-arm **PEG-BCN** was estimated to be 31−40 nm and the length size of **BPcSO_3_** was estimated to be 2−3 nm, based on which the mesh size of the click hydrogel was estimated to be 64−83 nm.

**2 Synthesis**

**Scheme S1**. Synthesis of 4-arm PEG-*exo*-BCN

**4**: This compound was synthesized following a reported procedure,^S^^[[2]](#endnote-3)^ and its identity was confirmed by comparing the obtained ^1^H NMR spectrum with the published one.

**5**: This compound was synthesized following a reported procedure,^S^^[[3]](#endnote-4)^ and its identity was confirmed by comparing the obtained ^1^H NMR spectrum with the published one.

^1^H NMR (400 MHz, Chloroform-*d*) *δ* 3.55 (dd, *J* = 6.3, 0.6 Hz, 2H), 2.42 (ddd, *J* = 13.7, 3.5, 1.8 Hz, 2H), 2.36 – 2.23 (m, 2H), 2.22 – 2.10 (m, 2H), 1.55 (s, 1H), 1.46 – 1.29 (m, 2H), 0.75 – 0.60 (m, 3H).

**1**: This compound was prepared following a reported procedure.^S^^[[4]](#endnote-5)^ To a stirred solution of **2** (170 mg, 1.1 mmol, 1.0 equiv.) in acetonitrile (5 ml) at room temperature, triethylamine (470 μl, 3.3 mmol, 3.0 equiv.) and NHS carbonate (570 mg, 1.6 mmol, 1.5 equiv.) were added sequentially. The reaction mixture was stirred at room temperature under nitrogen overnight. Upon completion, the solvent was removed, and the crude product was subject to column chromatography (hexane/EA=3:1 to 2:1) to yield a pale white solid (224 mg, 68%). m.p. 127.1 – 129.1 °C; ^1^H NMR (400 MHz, Chloroform-*d*) *δ* 4.26 (d, *J* = 6.9 Hz, 2H), 2.84 (s, 4H), 2.44 (dq, *J* = 13.5, 2.8 Hz, 2H), 2.37 – 2.25 (m, 2H), 2.24 – 2.10 (m, 2H), 1.47 – 1.35 (m, 2H), 0.91 – 0.75 (m, 3H); ^13^C NMR (101 MHz, Chloroform-*d*) δ 168.81, 151.81, 98.78, 77.46, 77.34, 77.14, 76.82, 76.12, 33.15, 25.61, 23.56, 22.99, 21.36;

**PEG-BCN**: 20 k 4-arm PEG-NH_2_ hydrochloride (400 mg, 0.08 mmol amine, 1.0 equiv.) and **2** (42 mg, 0.14 mmol, 7.0 equiv.) were dissolved in dimethylformamide (2.2 mL). *N*,*N*-Diisopropylethylamine (137.5 µL, 0.8 mmol, 10.0 equiv.) was added to the mixture, and the reaction was stirred for two days. The crude mixture was diluted with water and dialyzed using 1k MWCO dialysis tube (Spectra/Por® 6) against water for two days, followed by lyophilization to yield a white powder (360 mg, 85%). Functionalization was confirmed to be >95% by ^1^H-NMR by comparing integral values for characteristic BCN peaks (*δ* 2.39, 2.27) with those from the PEG backbone (*δ* 3.62).

**6**: This compound was synthesized following a reported procedure,^S^^[[5]](#endnote-6)^ and its identity was confirmed by comparing the obtained ^1^H NMR spectrum with the published one. It needs to be pointed out that the product after several rounds of column chromatography purification still contains *p*-nitro phenol, which is a degraded product from the starting material (*p*-nitro phenylchloroformate). The ‘crude’ compound (2.3 g) prepared from BODIPY-OH precursor (1.6 g) was hence used without further purification.

^1^H NMR (400 MHz, Chloroform-*d*) *δ* 8.31 – 8.24 (m, 2H), 7.44 – 7.34 (m, 2H), 5.58 (s, 2H), 2.51 (t, *J* = 1.1 Hz, 6H), 2.40 (q, *J* = 7.6 Hz, 4H), 2.35 (s, 6H), 1.05 (t, *J* = 7.6 Hz, 6H).

**2**: To a stirred solution of **4** (2.3 g, 4.6 mmol, 1 equiv.) in THF (115 ml) at room temperature, pyridine (368 μl, 4.6 mmol, 1 equiv.) and azido-PEG_3_-amine (1.6 g, 7.3 mmol, 1.6 equiv.) were added sequentially. The reaction mixture was stirred at 40 °C under nitrogen for 4 hours. Upon completion of the reaction monitored by TLC, water (100 ml) was added to quench the reaction, followed by extraction with DCM (100 ml × 3). The combined organic phase was washed with brine and dried over anhydrous Na_2_SO_4_. After removal of the solvent under reduced pressure, the crude product was subjected to column chromatography (EA/Heptane/DCM=1:2:1 to 1:1:1) to yield a dark red oil (2.3 g, 82% over two steps). ^1^H NMR (400 MHz, Chloroform-*d*) *δ* 5.51 (t, *J* = 4.9 Hz, 1H), 5.31 (s, 2H), 3.60 – 3.50 (m, 12H), 3.40 (q, *J* = 5.1 Hz, 2H), 3.26 (t, *J* = 4.8 Hz, 2H), 2.49 (s, 6H), 2.38 (q, *J* = 7.5 Hz, 4H), 2.29 (s, 6H), 1.04 (t, *J* = 7.6 Hz, 6H); ^13^C NMR (101 MHz, Chloroform-*d*) *δ* 156.08, 154.96, 136.88, 133.61, 132.35, 132.24, 70.73, 70.70, 70.66, 70.44, 70.07, 69.89, 58.46, 50.71, 41.18, 17.25, 14.84, 12.73; ^19^F NMR (376 MHz, Chloroform-*d*) *δ* -145.70 (dd, *J* = 65.8, 32.2 Hz); ESI-HRMS: *m/z* found [M+Na]^+^ for C_27_H_41_BF_2_N_6_O_5_Na^+^ 601.3095 (calcd. 601.3092).

**3**: To a stirred solution of **5** (1 g, 1.7 mmol, 1 equiv.) in DCM (30 ml) at room temperature, NBS (308 mg, 1.7 mmol, 1 equiv.) was added in one portion. The reaction mixture was stirred at room temperature for 40 min. After that, azido-PEG_3_-OH (1.9 g, 8.5 mmol, 5 equiv.) in DMF (15 ml) was added to the above solution and the reaction mixture was left stirring for another 4 hours. The reaction was then quenched with water (50 ml), followed by extraction with DCM (100 ml × 3). The combined organic phase was further washed with water (100 ml × 2) and brine, and dried over anhydrous Na_2_SO_4._ After removal of the solvent under reduced pressure, the crude product was subjected to column chromatography (DCM/Et_2_O/Heptane=1:2:1 to DCM/Et_2_O=1:2) to yield a dark red liquid (430 mg, 31%). ^1^H NMR (400 MHz, Chloroform-*d*) *δ* 5.51 (t, *J* = 5.6 Hz, 1H), 5.32 (s, 2H), 4.79 (s, 2H), 3.67 – 3.51 (m, 26H), 3.40 (q, *J* = 5.3 Hz, 2H), 3.36 (t, *J* = 5.6 Hz, 2H), 3.26 (t, *J* = 5.0 Hz, 2H), 2.54 – 2.49 (m, 5H), 2.39 (q, *J* = 7.7 Hz, 2H), 2.32 (s, 3H), 2.30 (s, 3H), 1.08 (t, *J* = 7.6 Hz, 3H) 1.05 (t, *J* = 16.7, 7.6 Hz, 3H); ^13^C NMR (101 MHz, Chloroform-*d*) δ 158.47, 155.99, 150.80, 138.77, 136.54, 135.02, 134.41, 133.72, 131.72, 70.82, 70.72, 70.68, 70.65, 70.46, 70.14, 70.09, 69.90, 69.68, 64.32, 58.36, 50.83, 50.72, 41.21, 17.33, 17.27, 15.06, 14.68, 13.12, 12.96, 12.46; ^19^F NMR (376 MHz, Chloroform-*d*) *δ* -141.25 (dd, *J* = 65.9, 32.0 Hz); ESI-HRMS: *m/z* found [M+Na]^+^ for C_35_H_56_BF_2_N_9_O_9_Na^+^ 818.4191 (calcd. 818.4154).

**BPcSO_3_**: To a stirred solution of **6** (42 mg, 0.05 mmol, 1 equiv.) in DCM (1.5 ml) in ice bath, NBS (13 mg, 0.08 mmol, 1.3 equiv.) was added in one portion. The reaction mixture was stirred at room temperature for 30 min. After that, MESNA (46 mg, 0.25 mmol, 5 equiv.) in DMF (2 ml) was added to the above solution and the reaction mixture was left stirring for another 2 hours. The crude was subjected to column chromatography (DCM/MeOH=20:1 to 12:1) to yield a dark red liquid (19 mg, 38%). ^1^H NMR (400 MHz, Methanol-*d*_4_) *δ* 5.43 (s, 2H), 4.83 (s, 2H), 4.12 (s, 2H), 3.82 – 3.49 (m, 28H), 3.40 – 3.36 (m, 4H), 3.07 (h, *J* = 1.6 Hz, 4H), 2.61 (qd, *J* = 7.5, 5.6 Hz, 4H), 2.44 (s, 3H), 2.44 (s, 3H), 1.20 (t, *J* = 7.5 Hz, 3H), 1.16 (t, *J* = 7.5 Hz, 3H); ^13^C NMR (101 MHz, Methanol-*d*_4_) *δ* 158.09, 156.64, 153.26, 141.07, 139.36, 137.15, 136.30, 134.54, 133.72, 71.61, 71.59, 71.49, 71.45, 71.42, 71.33, 71.29, 71.12, 71.07, 70.96, 70.76, 65.03, 59.21, 53.06, 51.78, 41.97, 28.70, 28.22, 18.11, 18.07, 15.36, 15.09, 13.03, 12.76; ^19^F NMR (376 MHz, Methanol-*d*_4_) *δ* -137.95 (dd, *J* = 65.7, 31.6 Hz); ESI-HRMS: *m/z* found [M-H]^-^ for C_37_H_58_BF_2_N_9_O_12_S_2_^-^ 934.3812 (calcd. 934.3791).

**3 NMR Characterization**


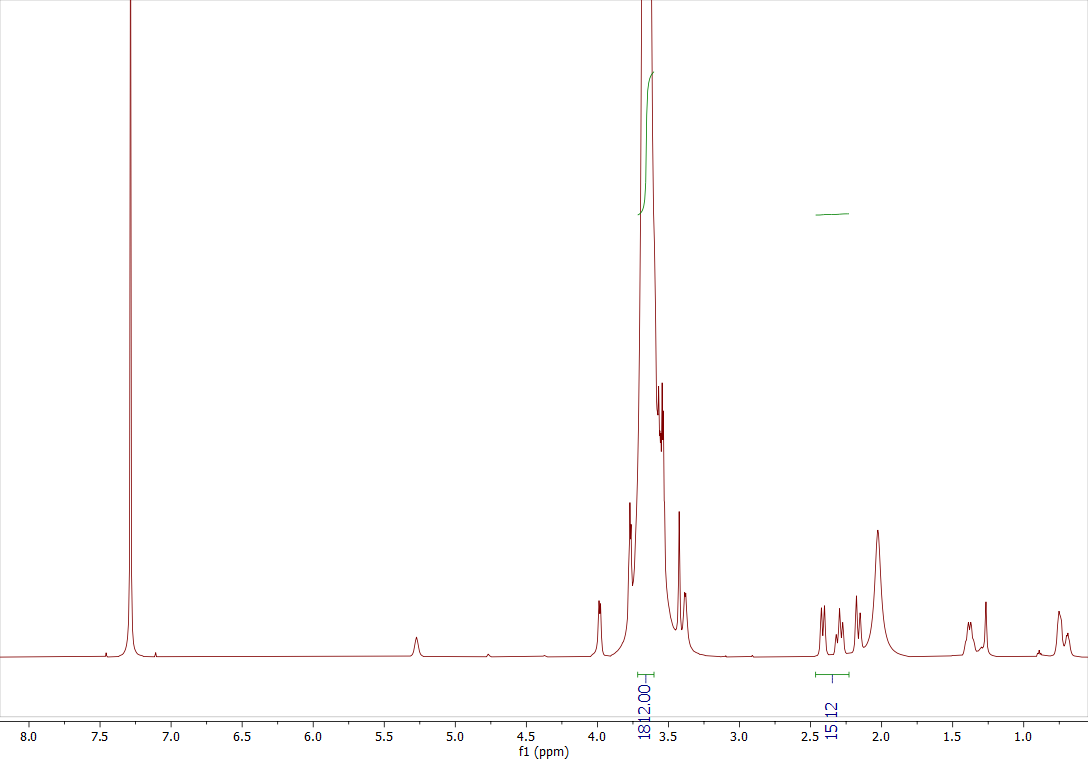


**Figure S1**. ^1^H NMR spectrum of **4-arm PEG-*exo*-BCN** in CDCl_3_, the degree of BCN functionalization is determined to be 95% based on the NMR integration.


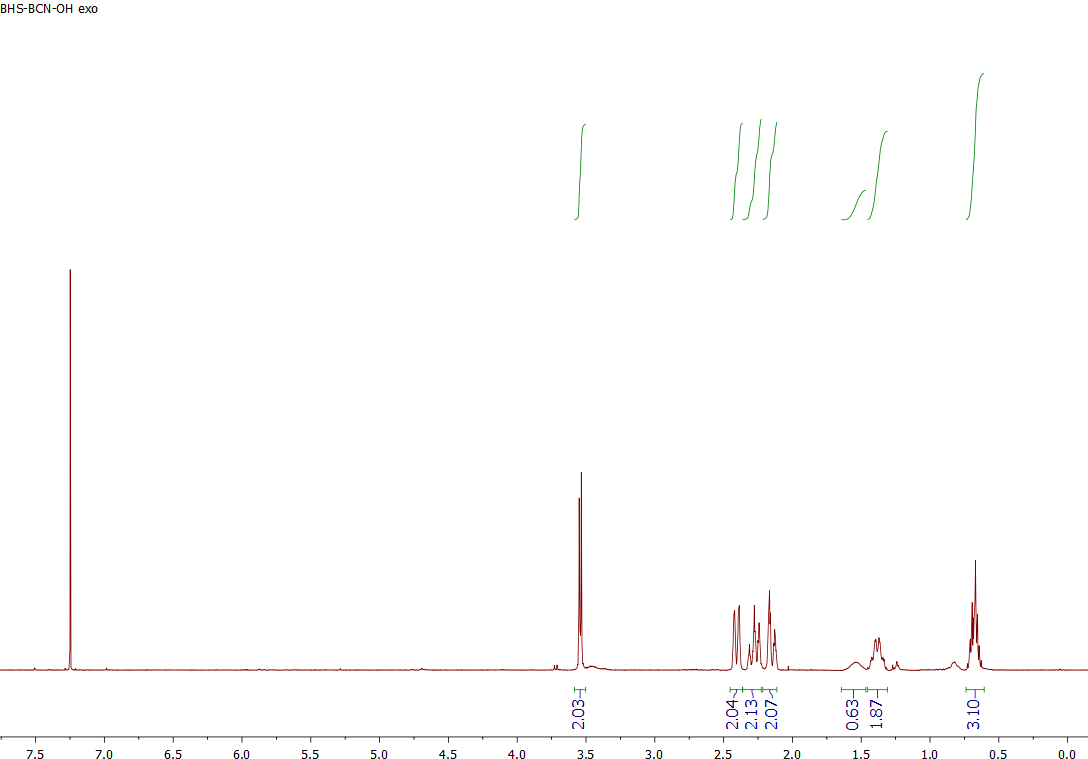


**Figure S2.** ^1^H NMR spectrum of **5** in CDCl_3_.


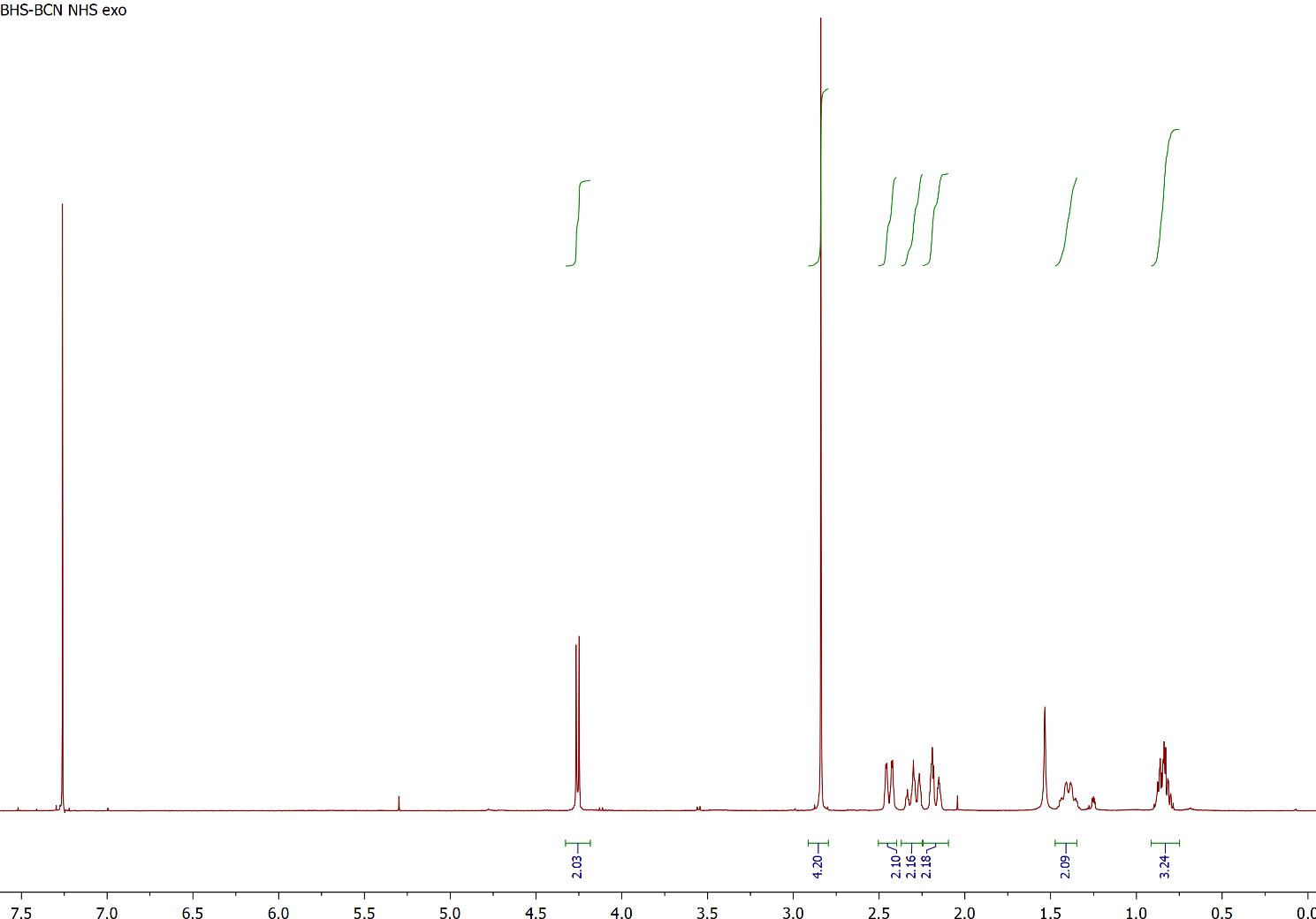


**Figure S3.** ^1^H NMR spectrum of **1** in CDCl_3_.


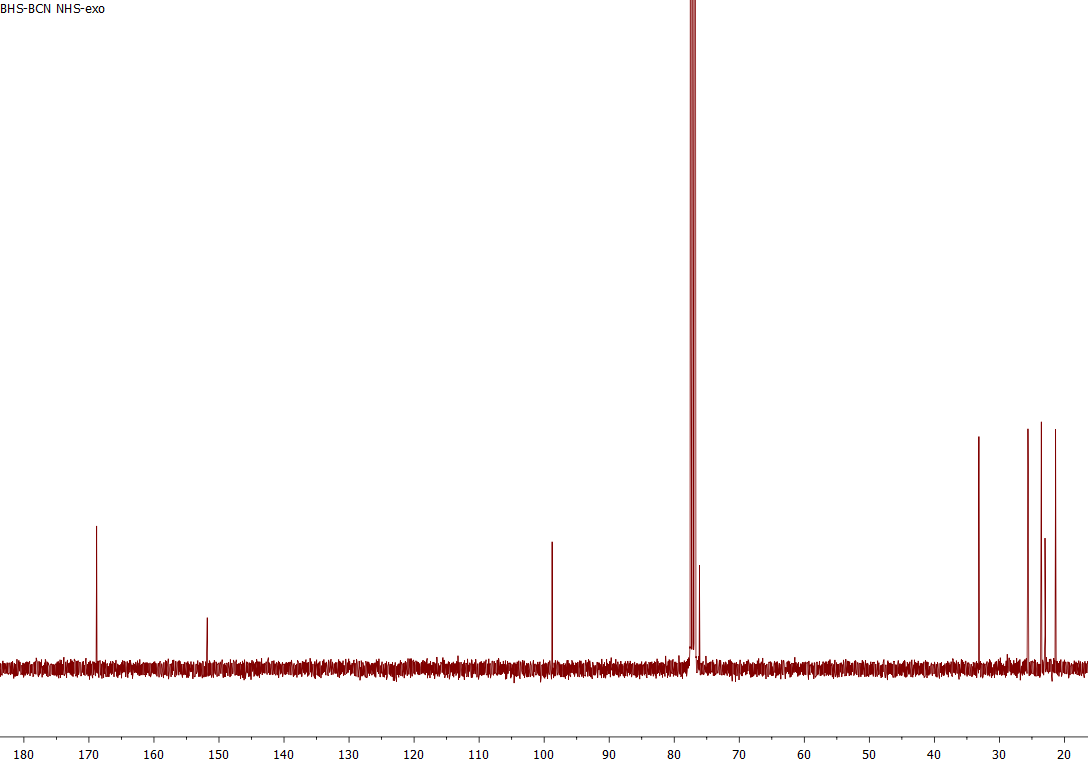


**Figure S4.** ^13^C NMR spectrum of **1** in CDCl_3_.


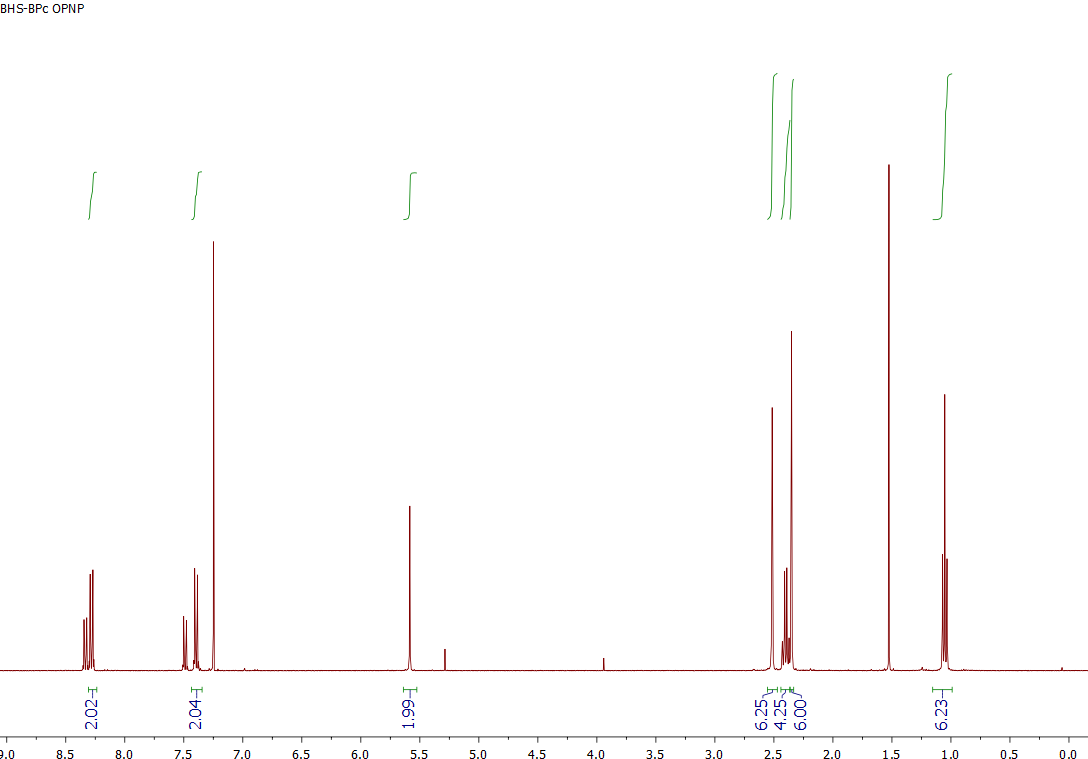


**Figure S5.** ^1^H NMR spectrum of **6** in CDCl_3_ (containing *p*-nitrophenol).


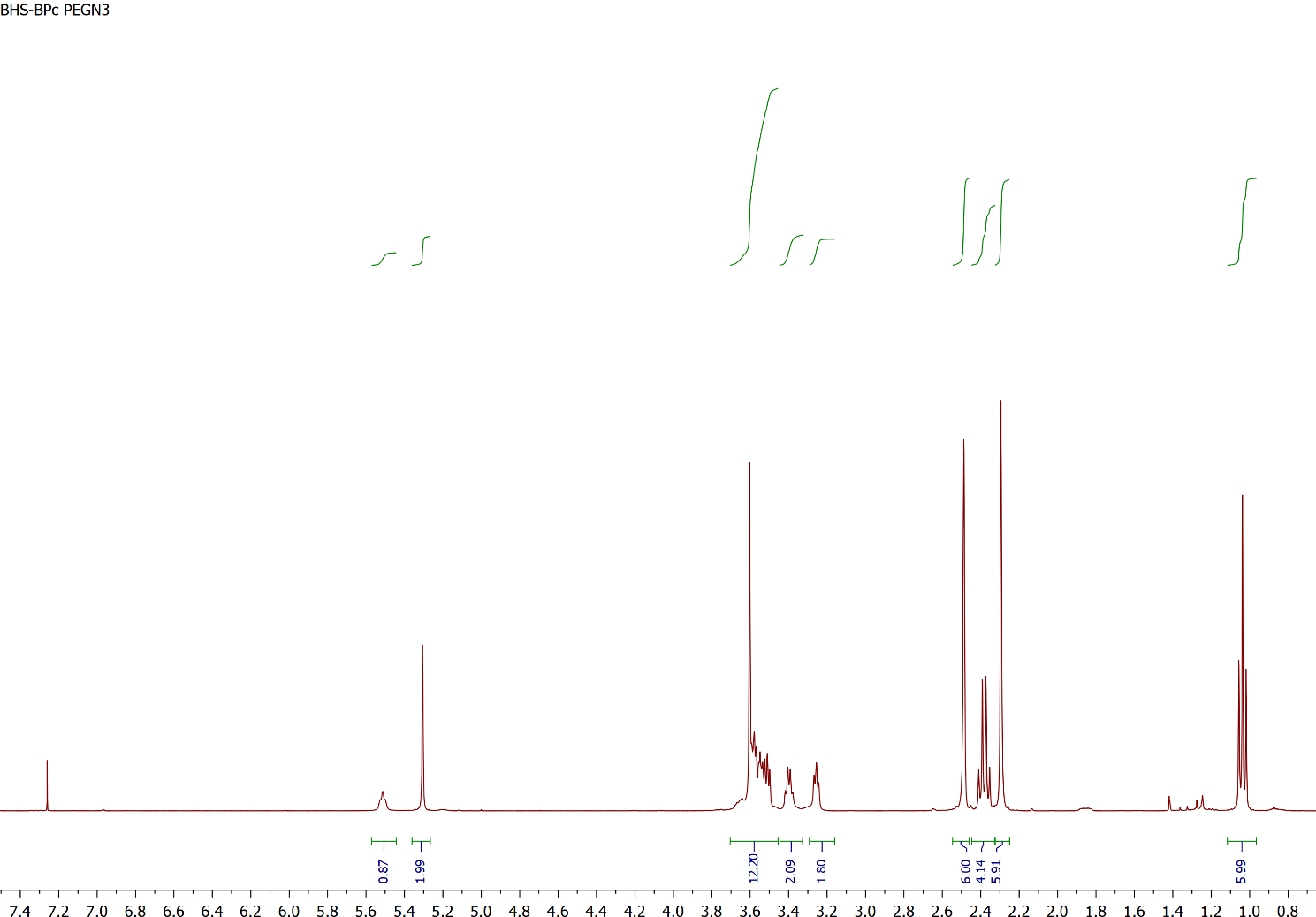


**Figure S6**. ^1^H NMR spectrum of **2** in CDCl_3_.


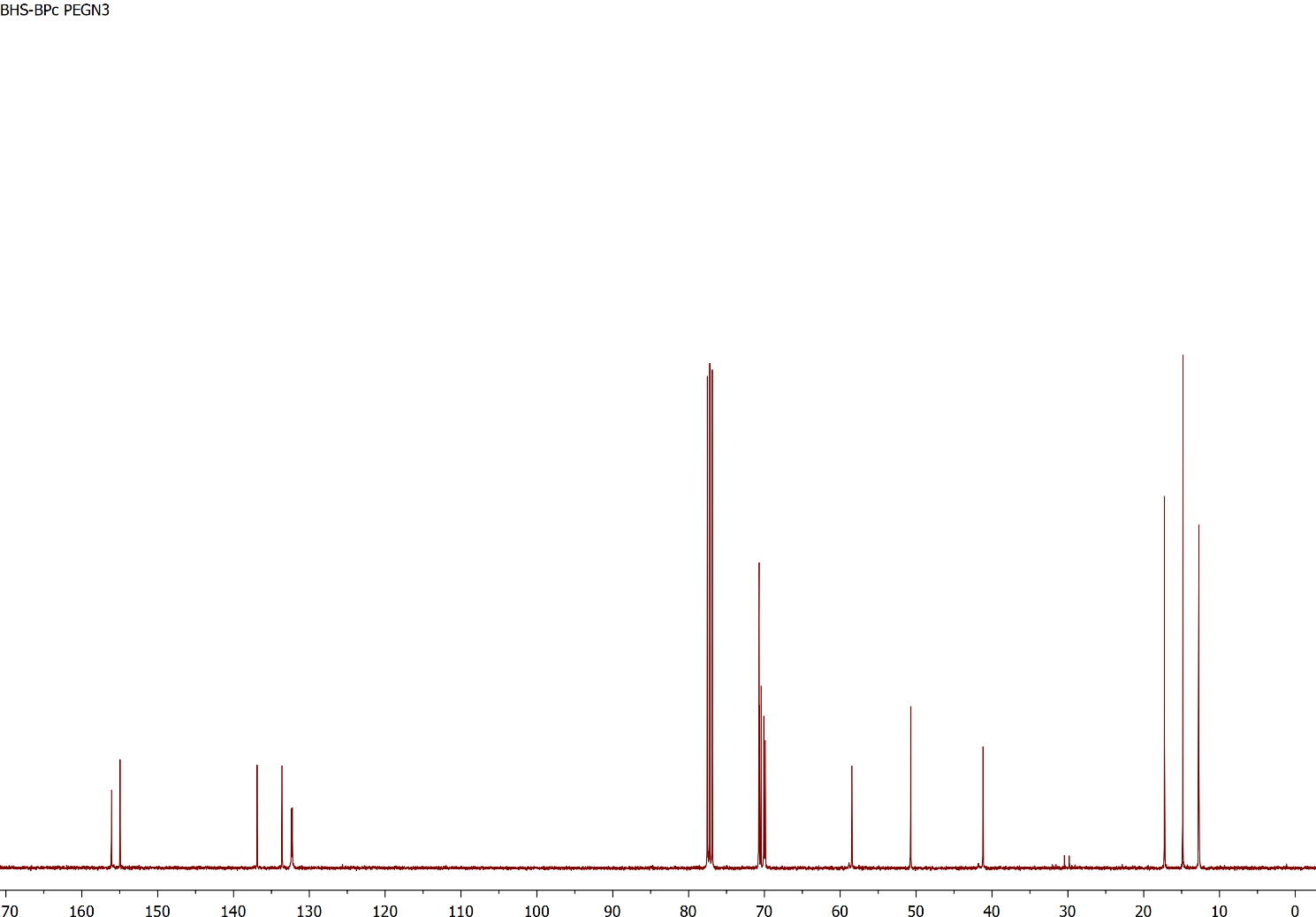


**Figure S7**. ^13^C NMR spectrum of **2** in CDCl_3_.


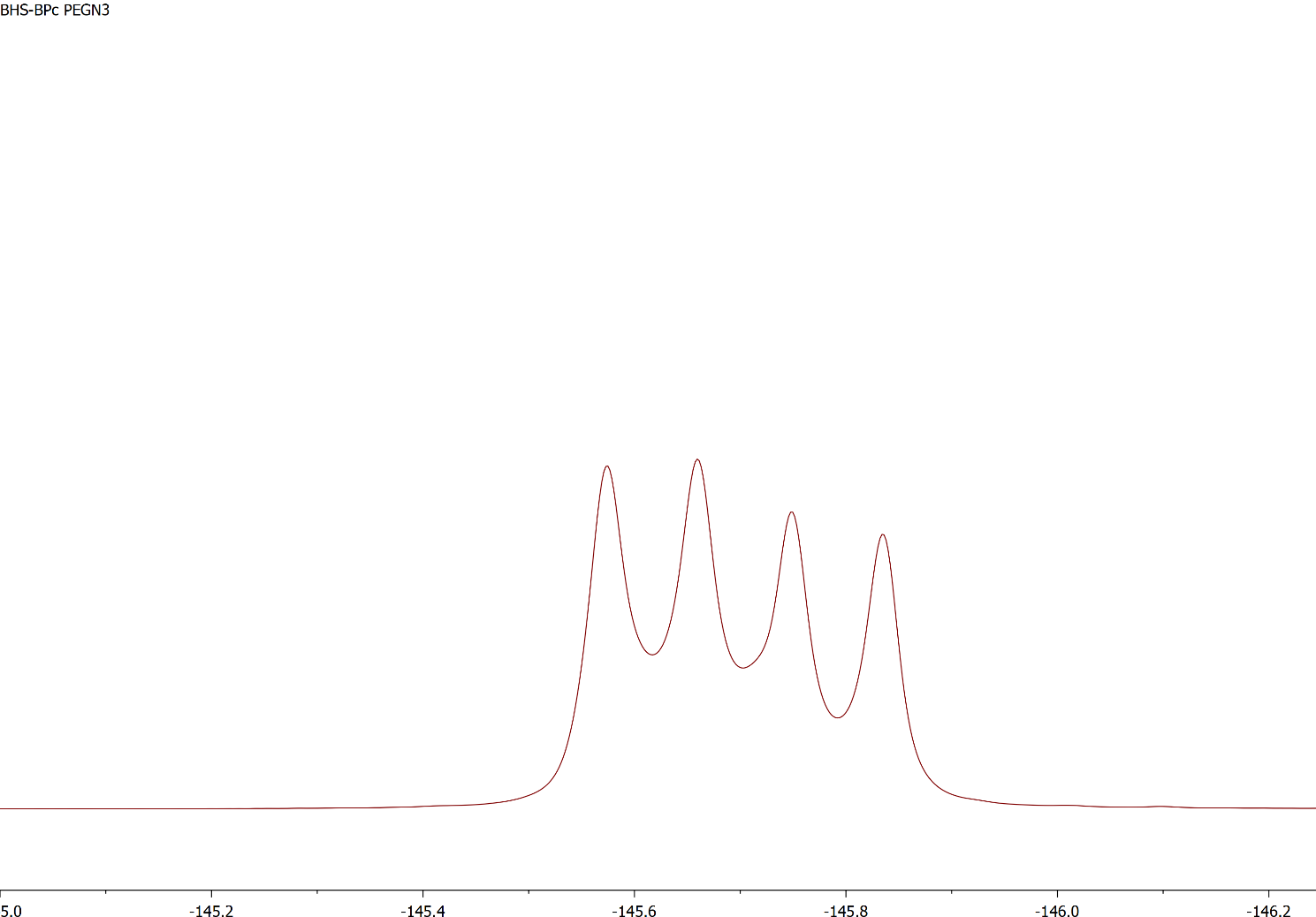


**Figure S8**. ^19^F NMR spectrum of **2** in CDCl_3_.


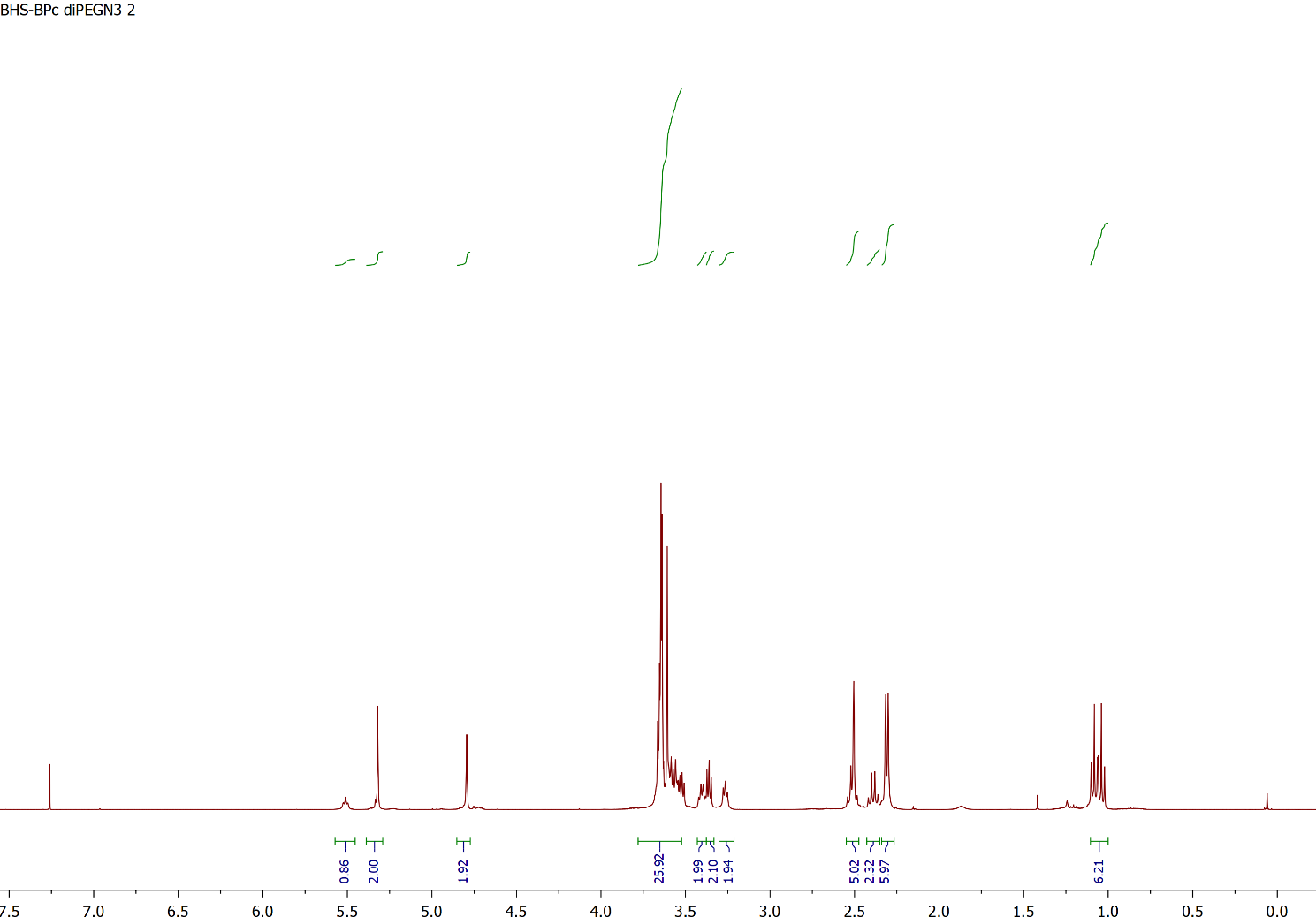


**Figure S9**. ^1^H NMR spectrum of **3** in CDCl_3_.


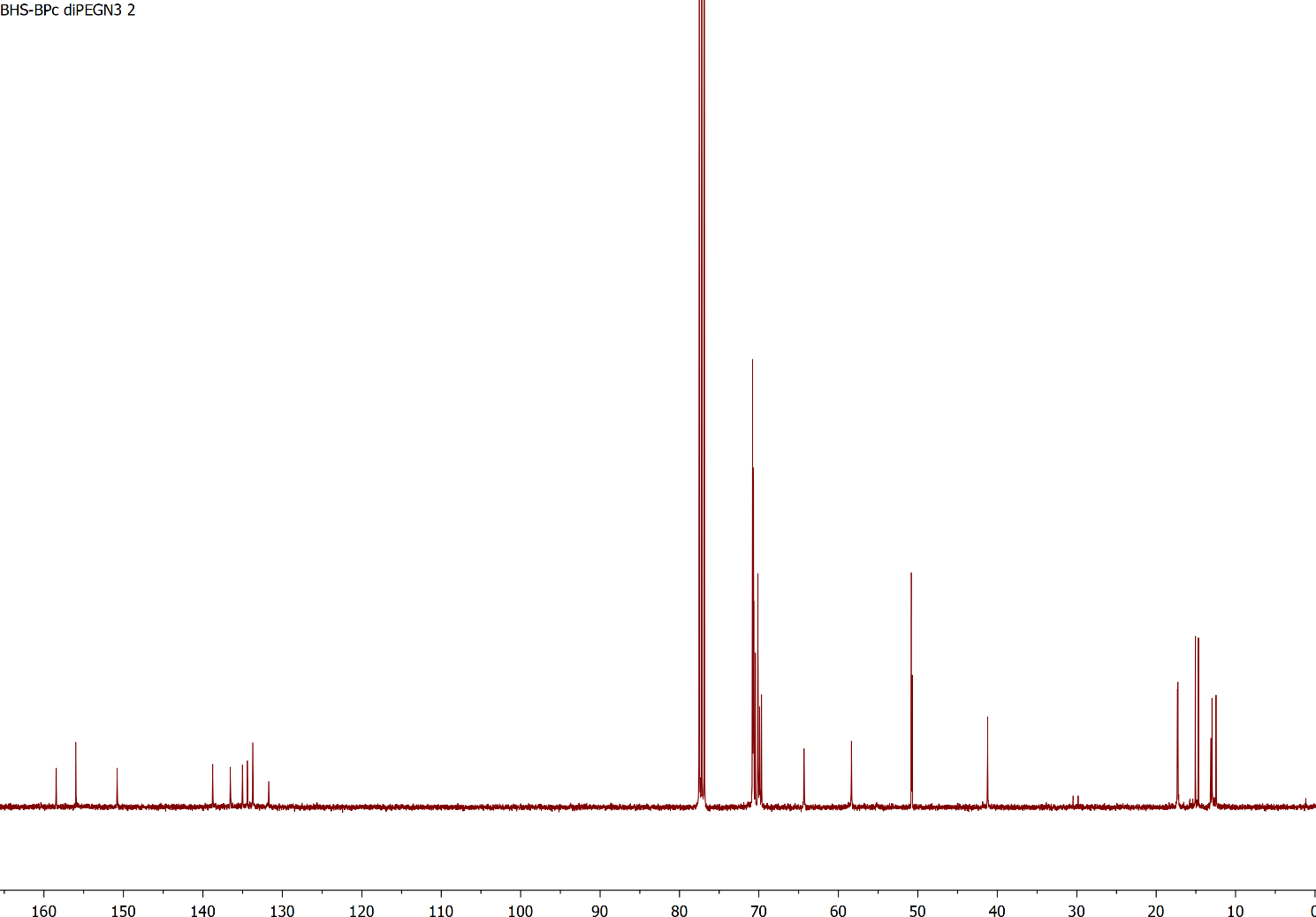


**Figure S10**. ^13^C NMR spectrum of **3** in CDCl_3_.


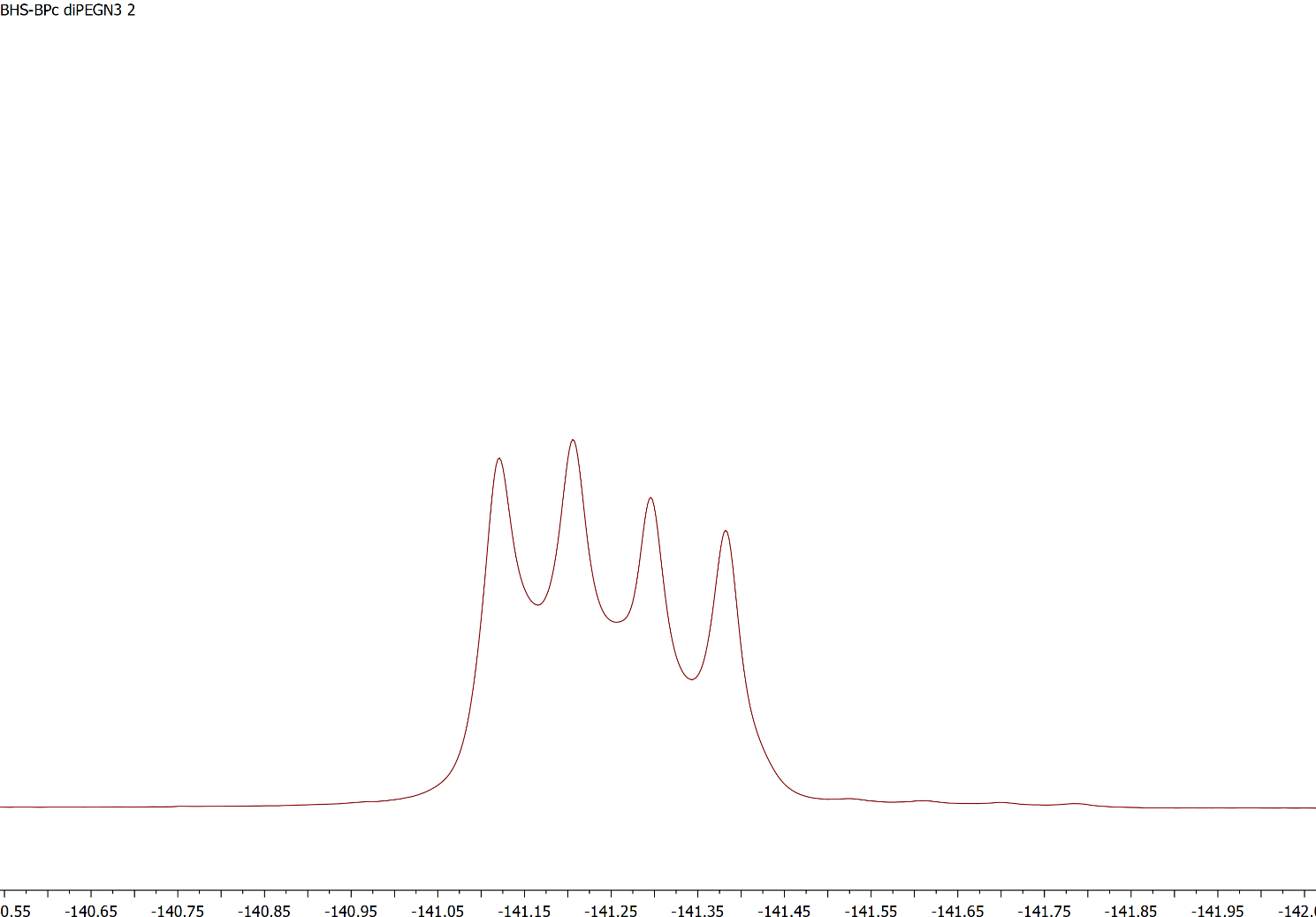


**Figure S11**. ^19^F NMR spectrum of **3** in CDCl_3_.


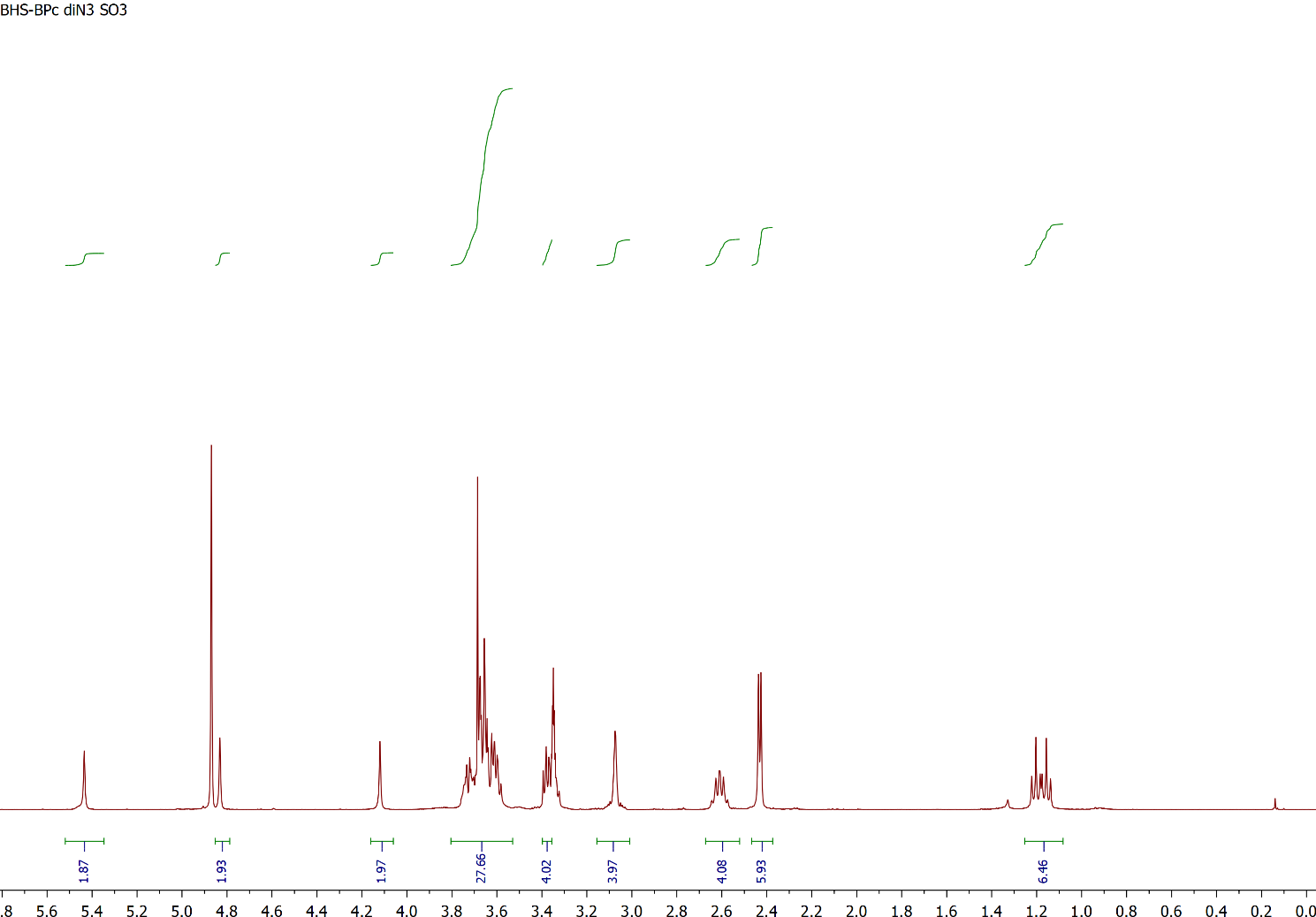


**Figure S12**. ^1^H NMR spectrum of **BPcSO_3_** in CD_3_OD.


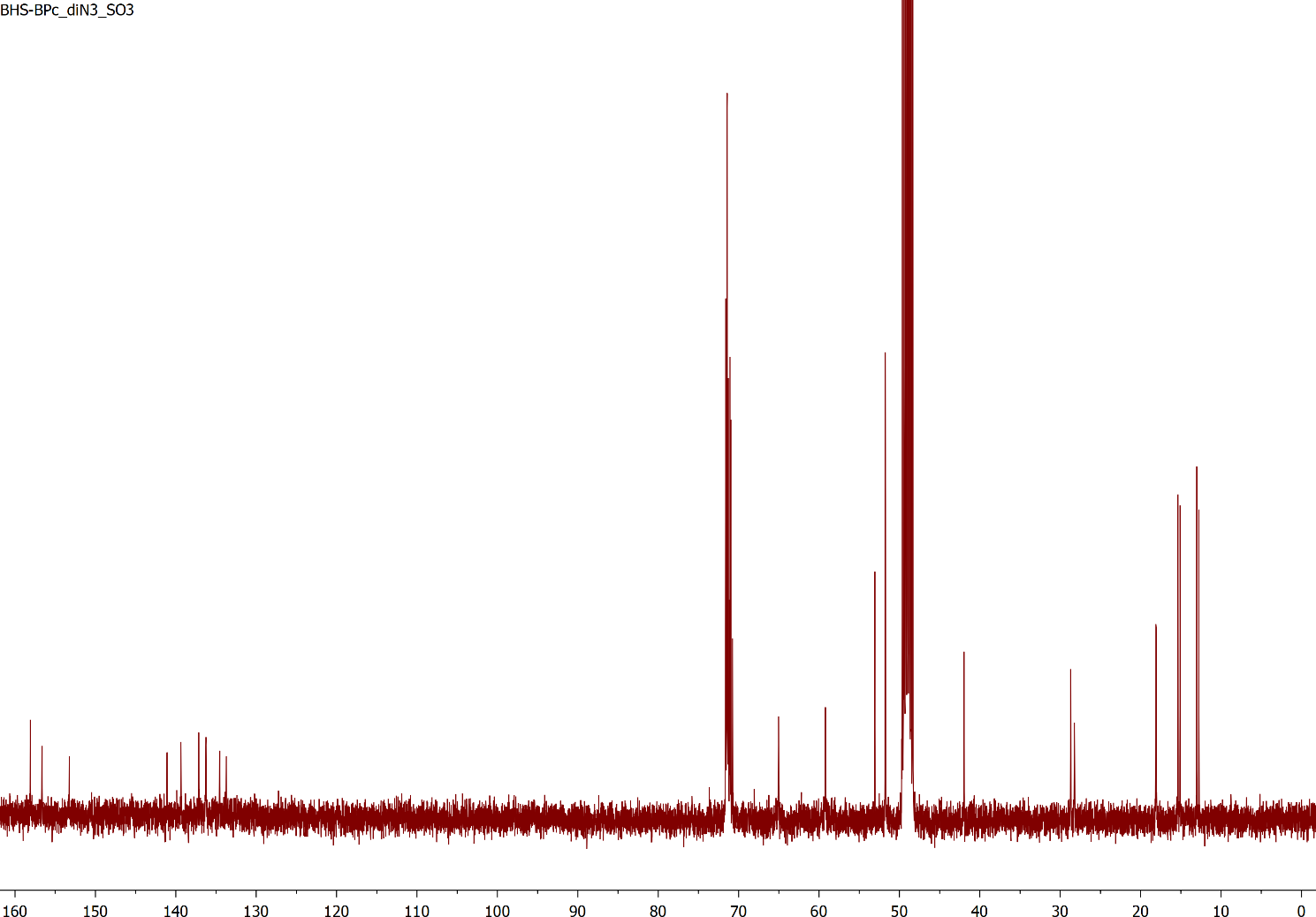


**Figure S13**. ^13^C NMR spectrum of **BPcSO_3_** in CD_3_OD.


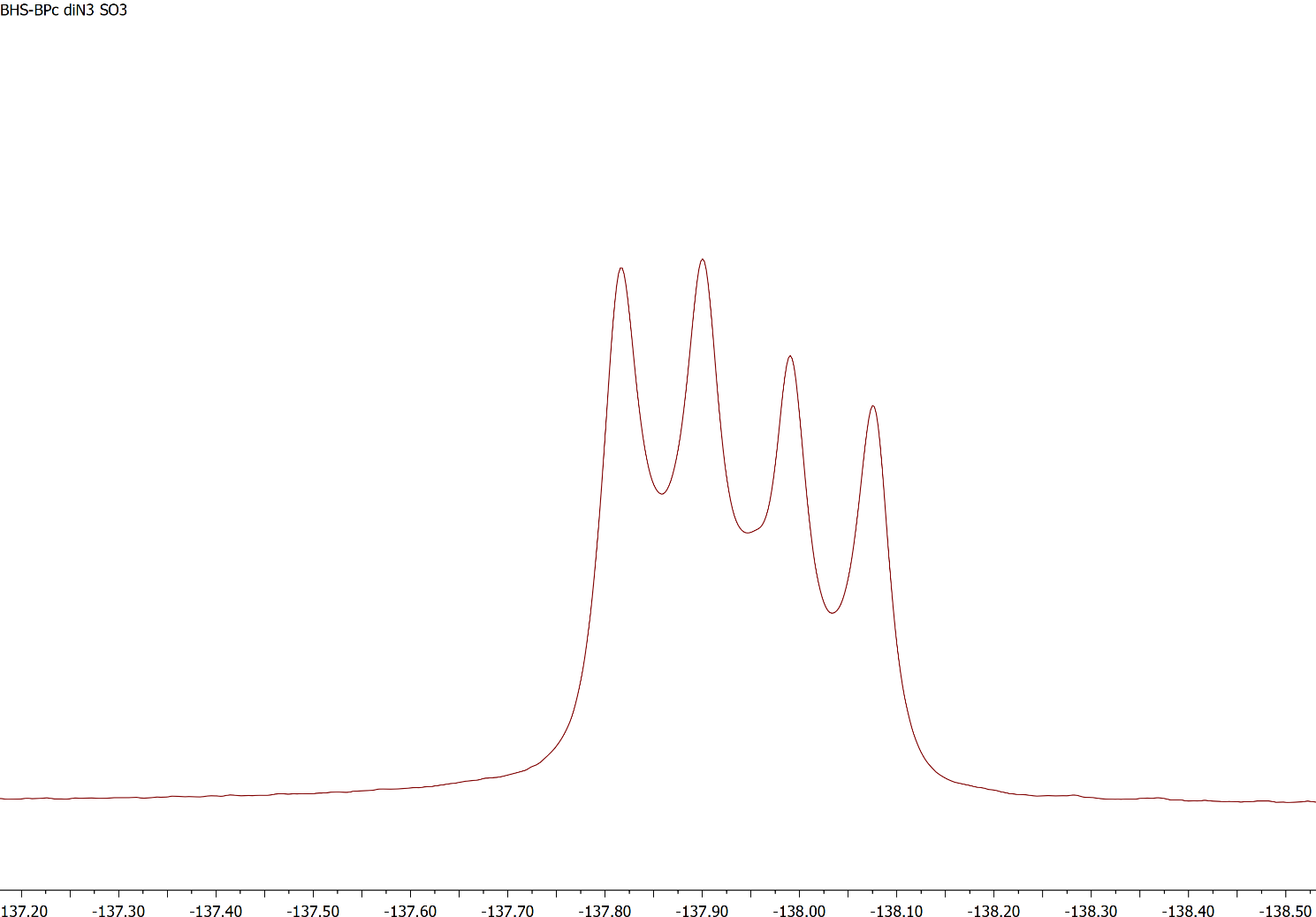


**Figure S14**. ^19^F NMR spectrum of **BPcSO_3_** in CD_3_OD.

**4 Characterization of the non-loaded hydrogels**

Time-, strain- and frequency-dependent oscillatory rheological experiments were performed on a rheometer (Antor-Paar MCR 302) with a stainless-steel measuring plate of 8 mm diameter and quartz plate with Peltier control (P-PTD 200/GL) at 298 K. Initial network formation kinetics of 1, 2, 3 and 4 wt% pre-gel solutions were monitored by following G′ and G″ at a constant strain (1%) and a frequency (5 rad/s) as a function of time.

**Figure S15**. Gel properties of 1 wt% samples were monitored *via* time-sweep experiments at fixed strain amplitude (1%) and frequency (5 rad/s) to measure G′ (Red) and G″ (Blue) of the hydrogels as a function of time at 298 K. Crossover timepoints were calculated from three repeated experiments.

**Figure S16**. Gel properties of 2 wt% samples were monitored *via* time-sweep experiments at fixed strain amplitude (1%) and frequency (5 rad/s) to measure G′ (Red) and G″ (Blue) of the hydrogels as a function of time at 298 K. Crossover timepoints were calculated from three repeated experiments.

**Figure S17**. Gel properties of 3 wt% samples were monitored *via* time-sweep experiments at fixed strain amplitude (1%) and frequency (5 rad/s) to measure G′ (Red) and G″ (Blue) of the hydrogels as a function of time at 298 K. Crossover timepoints were calculated from three repeated experiments.


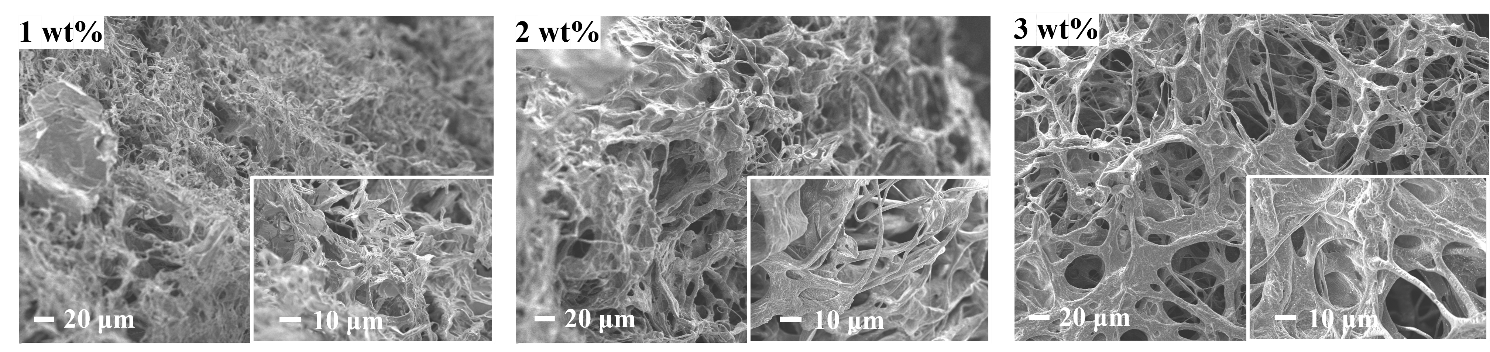


**Figure S18**. SEM images of the hydrogels at different wt%.

**Figure S19**. Cell viability for human dermal fibroblasts exposed to 4 wt% non-loaded hydrogels and light irradiation (*λ* = 530 nm; 15 mW/cm^2^) determined from an alamarBlue assay. Red: no gel was added while light was introduced; Blue: both gel and light were introduced; Green: gel was added while light was not introduced. Data shown as mean ± S.D. (N = 3, n = 1).

**5 Photo-uncaging quantum yield**

The photo-uncaging quantum yield (QY) was determined using **BPcOAc** (QY = 0.95%) as a reference photocage.^S^^[[6]](#endnote-7)^ A 20 μM solution of BPcOAc or **BPcSO_3_** in DMSO/H2O (1:10, v/v) were prepared and transferred to a 96-well quartz plate. A green LED array (*λ* = 530 nm; 15 mW/cm2) was used to irradiate the samples and the absorbance maxima of **BPcOAc** (*λ* = 540 nm) and **BPcSO_3_** (*λ* = 550 nm) were recorded and plotted as a function of time. Photo-uncaging QY (%) = (photo-uncaging rate of **BPcSO_3_** / photo-uncaging rate of **BPcOAc**) × photo-uncaging QY of **BPcOAc** × 100 %. The QY determination was carried out in triplicate.


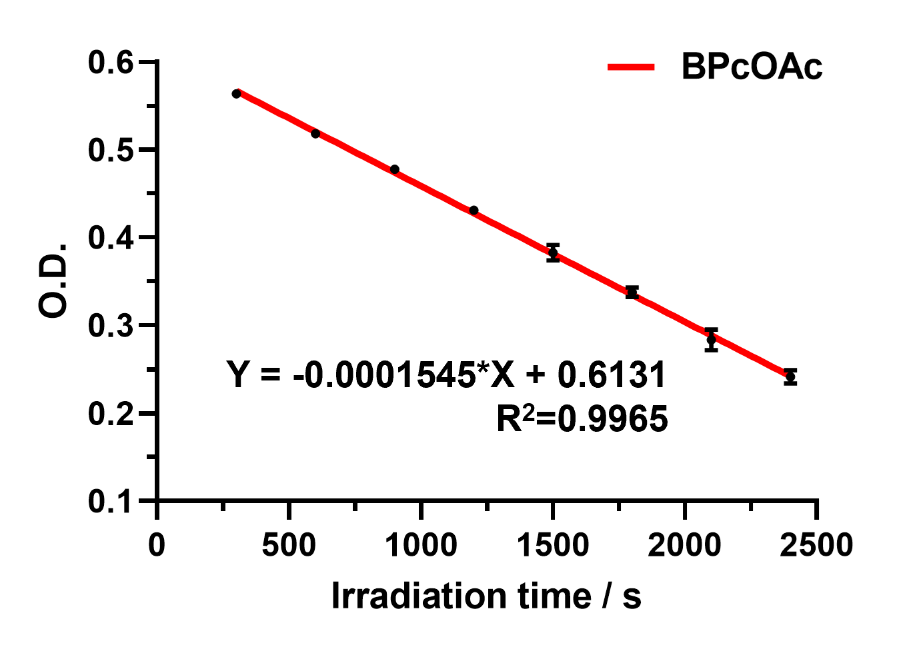


**Figure S20**. Photo-uncaging kinetics of **BPcOAc** (20 μM) in DMSO/H_2_O (1:10, v/v) under irradiation of a green LED light array (*λ* = 530 nm; 15 mW cm^-2^). The plot is the absorbance of **BPcOAc** (*λ* = 540 nm) as a function of irradiation time. Data are presented as mean ± s.d. (n = 3).


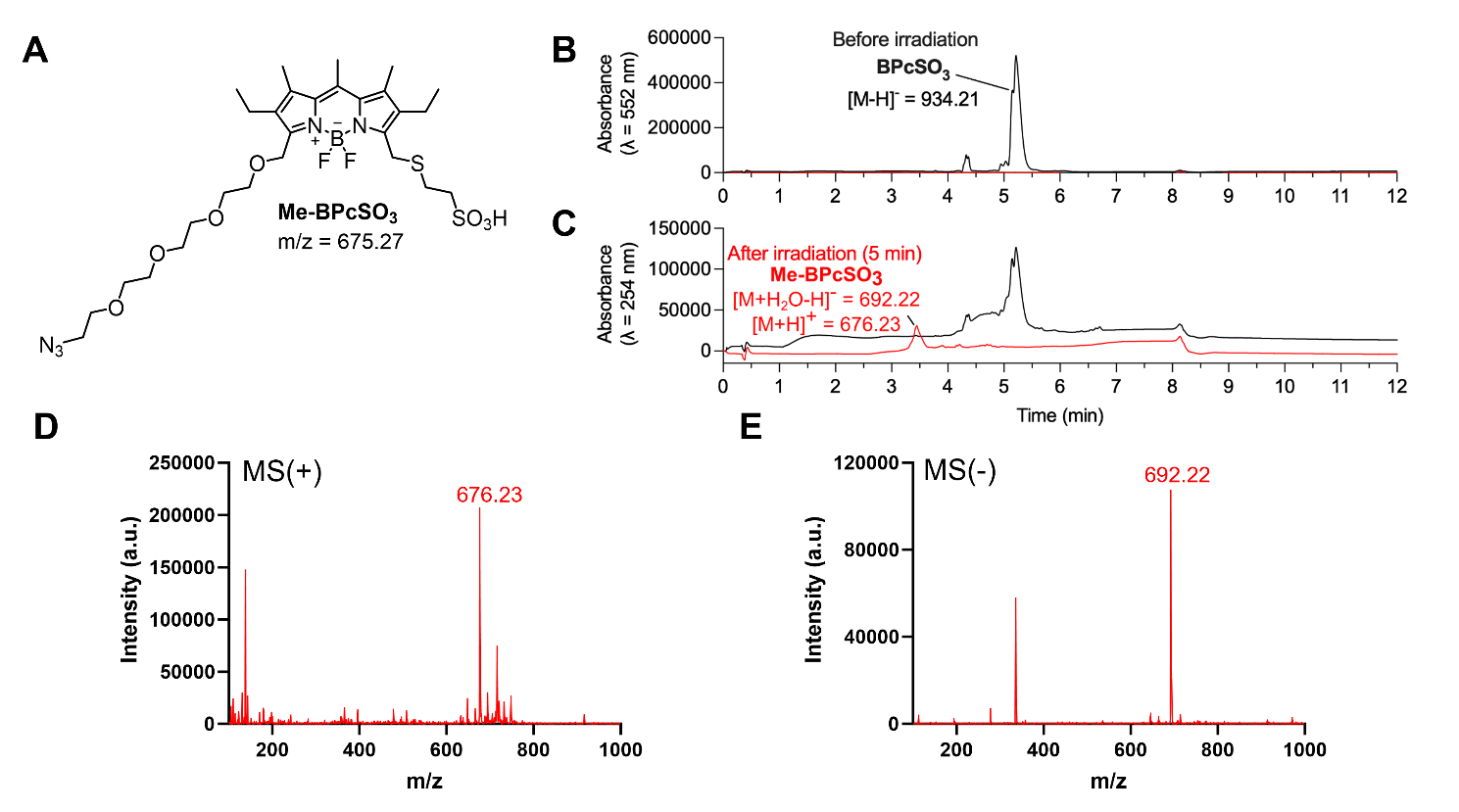


**Figure S21**. The proposed photo-uncaged product **Me-BPcSO_3_** (**A**) and LC-MS chromatograms of **BPcSO_3_** (20 μM, 500 μL) in water before (**B**) and after irradiation (**C**, *λ* = 530 nm; 15 mW cm^-2^). After 5 minutes of irradiation, **BPcSO_3_** is completely photo-uncaged as determined by consumption of the **BPcSO_3_** in the HPLC chromatogram. The LC traces include the peaks corresponding to the observed m/z values (**D**, **E**).

**6 Characterization of the control hydrogels**

PBS solution of PEG-diazide (M_n_ = 450; 90 µM) was prepared from a 0.1 g/mL THF stock. 4 wt% gel samples were obtained from rapid mixing of **PEG**-**BCN** and PEG-diazide solutions which were left to gelate for 2 hours before use. Once the gels had been formed, PBS was added, and gel pellets were gently detached from the mould using a thin pipette tip. Gels were then kept in a humid environment before measurements.

**Figure S22**. Gel properties of 4 wt% control gels were monitored *via* time-sweep experiments at fixed strain amplitude (1%) and frequency (5 rad/s) to measure G′ (red) and G″ (blue) of the hydrogels as a function of time at 298 K. Crossover timepoints were calculated from three repeated experiments.

**Figure S23**. Gel properties of 4 wt% control gels were monitored *via* strain-sweep experiments at a fixed frequency (10 rad/s) to measure G′ (red) and G″ (blue) as a function of strain amplitude.

**Figure S24**. Gel properties of 4 wt% control gels were monitored *via* frequency-sweep experiments at a fixed strain (0.1%) to measure G′ (red) and G″ (blue) as a function of frequency.

**Figure S25**. Light-triggered gel degradation of 4 wt% control gel under light irradiation (*λ* = 550 nm). The plot shows the changes in G′ of the gels as a function of irradiation time.

**7 Characterization of the PNSP-loaded hydrogels**

Gel samples were obtained from rapid mixing of **PEG**-**BCN**, **BPcSO_3_** and PSNP solutions at different ratios, which were left to gelate for 2 hours before use. Once the gels had been formed, PBS was added, and gel pellets were gently detached from the mould using a thin pipette tip. Gels were then kept in a humid environment before measurements.

**Table S1.** Summary of different Gel-PSNP formulations

| Gel-PSNP formulation | Volume of 4wt% gel [µL] | Volume of 50 mg/ml PSNP [µL] | wt% of Gel-PSNP [%] | Concentration of PSNP [mg/ml] |
| --- | --- | --- | --- | --- |
| 25% | 40 | 10 | 4.2 | 10.0 |
| 50% | 40 | 20 | 4.3 | 16.7 |
| 75% | 40 | 30 | 4.4 | 21.4 |
| 100% | 40 | 40 | 4.5 | 25.0 |

**Figure S26**. Gel properties of 4 wt% + PSNP (100 nm; 25%) samples were monitored *via* strain-sweep experiments at a fixed frequency (10 rad/s) to measure G′ (red) and G″ (blue) as a function of strain amplitude.

**Figure S27**. Gel properties of 4 wt% + PSNP (100 nm; 25%) samples were monitored *via* frequency-sweep experiments at a fixed strain (0.1%) to measure G′ (red) and G″ (blue) as a function of frequency.

**Figure S28**. Gel properties of 4 wt% + PSNP (100 nm; 50%) samples were monitored *via* strain-sweep experiments at a fixed frequency (10 rad/s) to measure G′ (red) and G″ (blue) as a function of strain amplitude.

**Figure S29**. Gel properties of 4 wt% + PSNP (100 nm; 50%) samples were monitored *via* frequency-sweep experiments at a fixed strain (0.1%) to measure G′ (red) and G″ (blue) as a function of frequency.

**Figure S30**. Gel properties of 4 wt% + PSNP (100 nm; 75%) samples were monitored *via* strain-sweep experiments at a fixed frequency (10 rad/s) to measure G′ (red) and G″ (blue) as a function of strain amplitude.

**Figure S31**. Gel properties of 4 wt% + PSNP (100 nm; 75%) samples were monitored *via* frequency-sweep experiments at a fixed strain (0.1%) to measure G′ (red) and G″ (blue) as a function of frequency.

**Figure S32**. Gel properties of 4 wt% + PSNP (100 nm; 100%) samples were monitored *via* strain-sweep experiments at a fixed frequency (10 rad/s) to measure G′ (red) and G″ (blue) as a function of strain amplitude.

**Figure S33**. Gel properties of 4 wt% + PSNP (100 nm; 100%) samples were monitored *via* frequency-sweep experiments at a fixed strain (0.1%) to measure G′ (red) and G″ (blue) as a function of frequency.

**8 Characterization of the ovalbumin-loaded hydrogels**

Gel samples were obtained from rapid mixing of **PEG**-**BCN**, **BPcSO_3_** and Ovalbumin solutions at different ratios, which were left to gelate for 2 hours before use. Once the gels had been formed, PBS was added, and gel pellets were gently detached from the mould using a thin pipette tip. Gels were then kept in a humid environment before measurements.

**Table S2.** Summary of different Gel-Ova formulations

| Gel-Ova formulation | Volume of 4wt% gel [µL] | Volume of 40 mg/ml Ova [µL] | wt% of Gel-Ova [%] | Concentration of Ova [mg/ml] |
| --- | --- | --- | --- | --- |
| 25% | 40 | 10 | 4.0 | 8.0 |
| 50% | 40 | 20 | 4.0 | 13.3 |
| 75% | 40 | 30 | 4.0 | 17.1 |
| 100% | 40 | 40 | 4.0 | 20.0 |

**Figure S34**. Gel properties of 4 wt% + Ovalbumin (25%) samples were monitored *via* strain-sweep experiments at a fixed frequency (10 rad/s) to measure G′ (red) and G″ (blue) as a function of strain amplitude.

**Figure S35**. Gel properties of 4 wt% + Ovalbumin (25%) samples were monitored *via* frequency-sweep experiments at a fixed strain (0.1%) to measure G′ (red) and G″ (blue) as a function of frequency.

**Figure S36**. Gel properties of 4 wt% + Ovalbumin (50%) samples were monitored *via* strain-sweep experiments at a fixed frequency (10 rad/s) to measure G′ (red) and G″ (blue) as a function of strain amplitude.

**Figure S37**. Gel properties of 4 wt% + Ovalbumin (50%) samples were monitored *via* frequency-sweep experiments at a fixed strain (0.1%) to measure G′ (red) and G″ (blue) as a function of frequency.

**Figure S38**. Gel properties of 4 wt% + Ovalbumin (75%) samples were monitored *via* strain-sweep experiments at a fixed frequency (10 rad/s) to measure G′ (red) and G″ (blue) as a function of strain amplitude.

**Figure S39**. Gel properties of 4 wt% + Ovalbumin (75%) samples were monitored *via* frequency-sweep experiments at a fixed strain (0.1%) to measure G′ (red) and G″ (blue) as a function of frequency.

**Figure S40**. Gel properties of 4 wt% + Ovalbumin (100%) samples were monitored *via* strain-sweep experiments at a fixed frequency (10 rad/s) to measure G′ (red) and G″ (blue) as a function of strain amplitude.

**Figure S41**. Gel properties of 4 wt% + Ovalbumin (100%) samples were monitored *via* frequency-sweep experiments at a fixed strain (0.1%) to measure G′ (red) and G″ (blue) as a function of frequency.


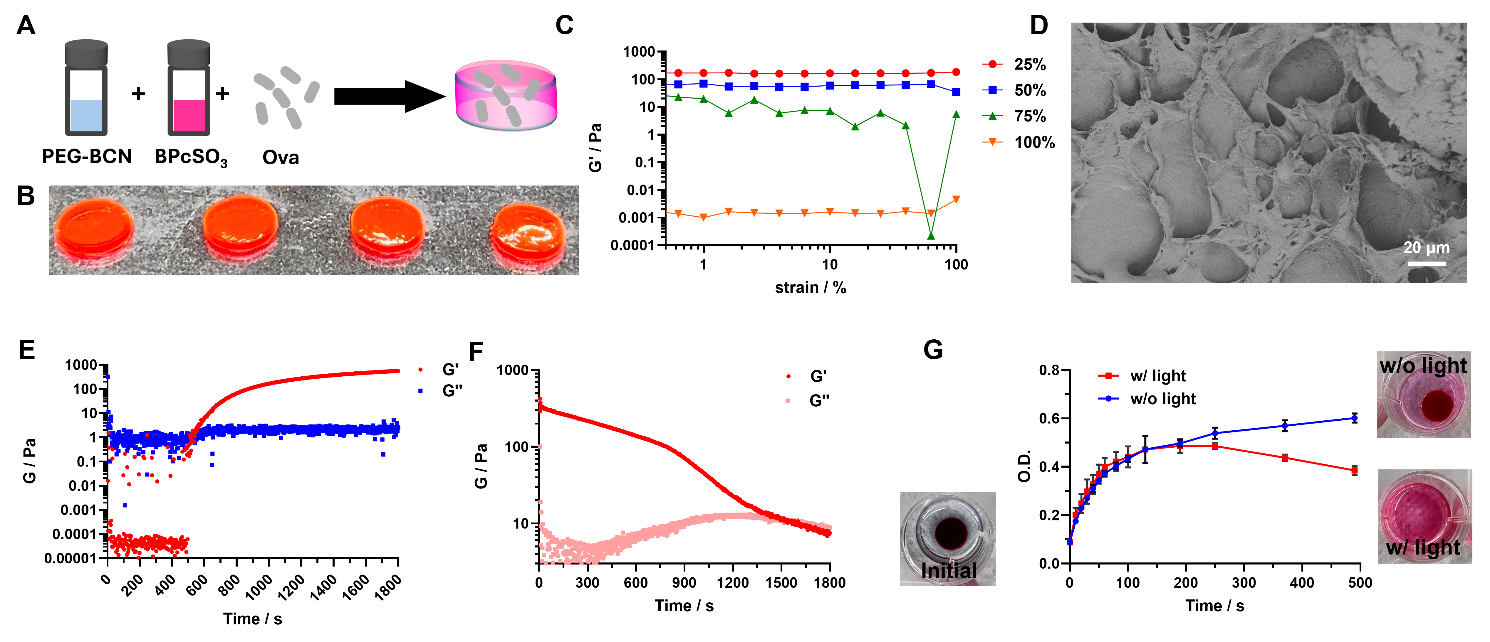


**Figure S42.** Encapsulation and photo-release of ovalbumin in 4 wt% hydrogels. **A**) Gel preparation from mixing PBS solutions of **PEG**-**BCN**, **BPcSO_3_** and ovalbumin. **B**) Image of the discs of different hydrogel-protein composites demolded from 8-millimetre PDMS molds, from left to right, representing the composites containing 25, 50, 75 and 100% of ovalbumin, respectively. **C**) Gel properties of different hydrogel-protein composites were monitored *via* strain-sweep experiments at a fixed frequency (10 rad/s) to measure G′ (red) and G″ (blue) as a function of strain amplitude. **D**) SEM image of the hydrogel-protein composite (25%). **E**) Gel properties of the hydrogel-protein composite (25%) were monitored *via* time-sweep experiments at fixed strain amplitude (10%) and frequency (5 rad/s) to measure G′ (red) and G″ (blue) of the composites as a function of time at 298 K. **F**). Light-triggered degradation of the hydrogel-protein composite (25%) under light irradiation (*λ* = 530 nm; 163 mW/cm^2^). The plot shows the change in G′ of the gel as a function of irradiation time. **G**) Light-controlled release of ovalbumin from the composites, where the inset images show the gel suspensions with or without light treatment. Absorbance (*λ* = 650 nm) of CF647-labelled ovalbumin was monitored as a function of irradiation time. (N = 3).


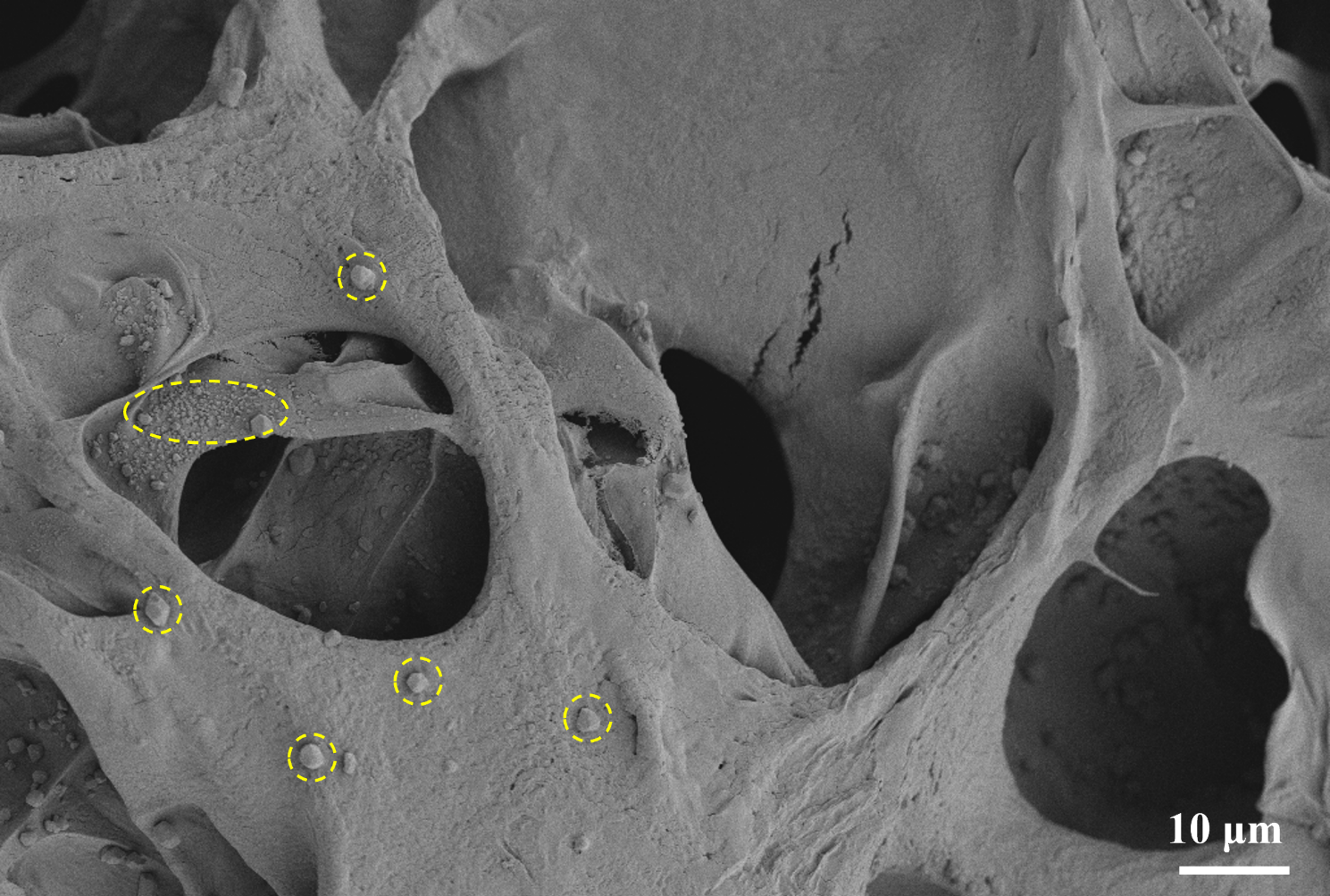


**Figure S43.** SEM image of the hydrogel-protein composite (25%). Representative protein aggregates were yellow circled.

**9 Injection force measurement**

**Figure S44.** Injection force *versus* time for 100 µL 4 wt% hydrogel, after 5 minutes of mixing, injected from a 1 mL syringe with a 21 G needle.

**Figure S45.** Injection force *versus* time for 100 µL 4 wt% hydrogel, after 5 minutes of mixing, injected from a 1 mL syringe with a 23 G needle.

**10 *Ex vivo* demonstration in porcine tissues and human skin explants**

**Figure S46.** Normalized volume of the gel samples retrieved from porcine tissues without (red) or with (blue) light treatments. The relative gel size was determined from the retrieved gels in Figure 5E using ImageJ.


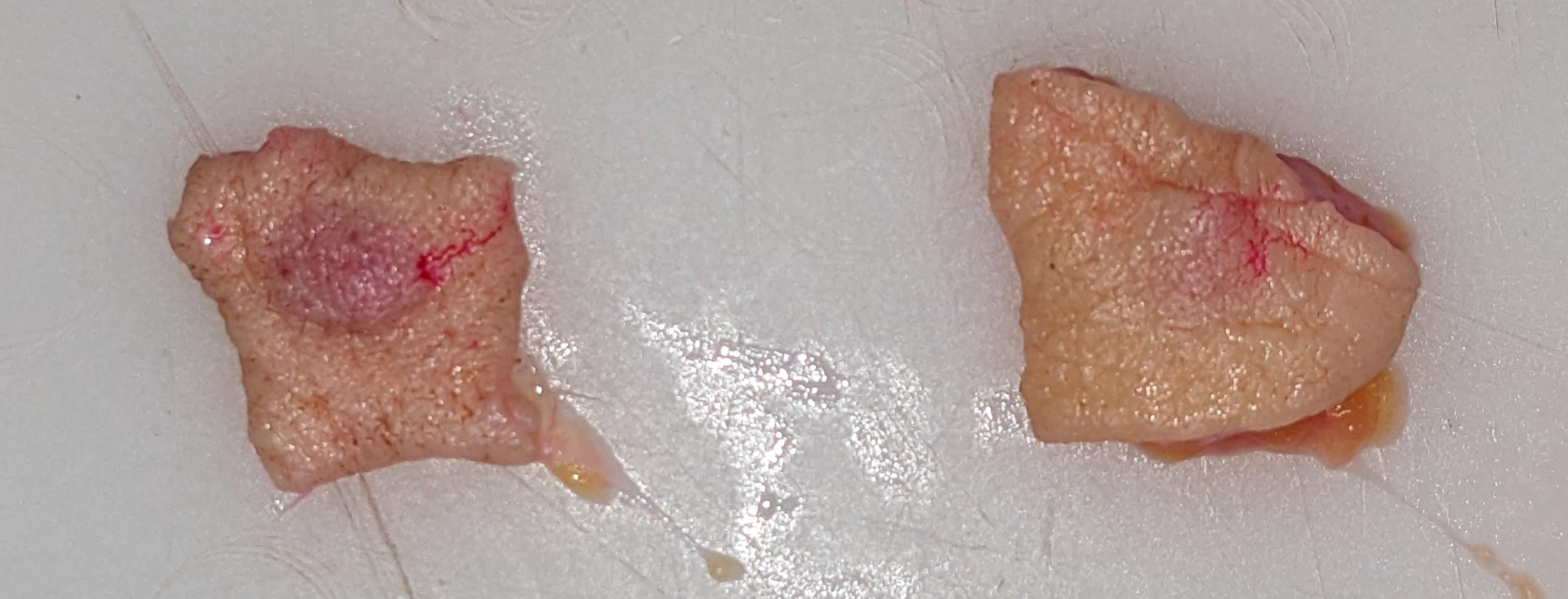

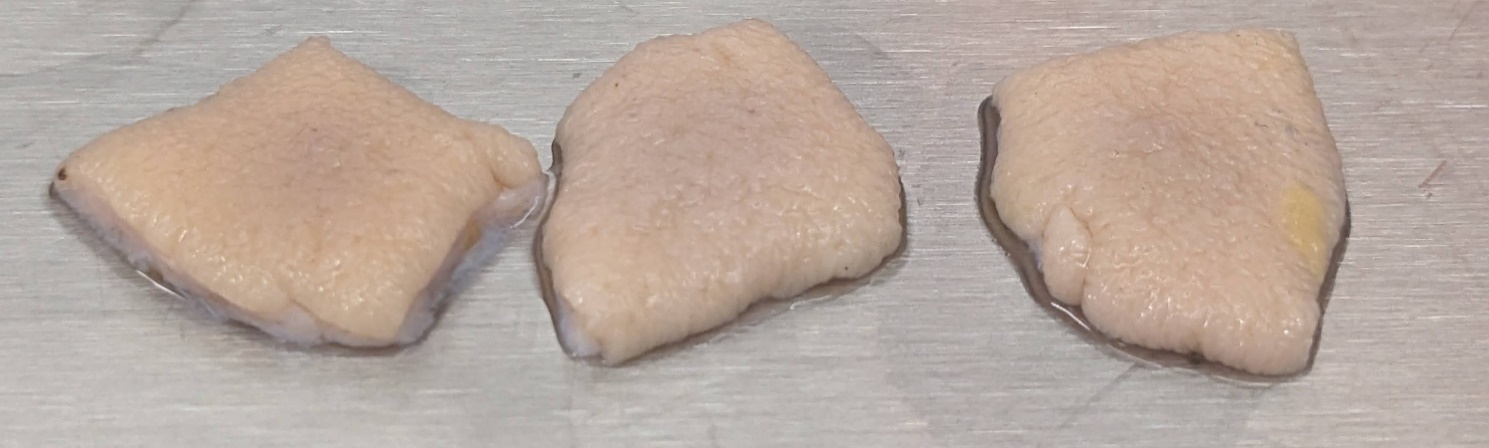


**Figure S47.** Photographs of human skin explants (~1 cm^3^) immediately (left) and seven days (right) after intradermal gel injection.


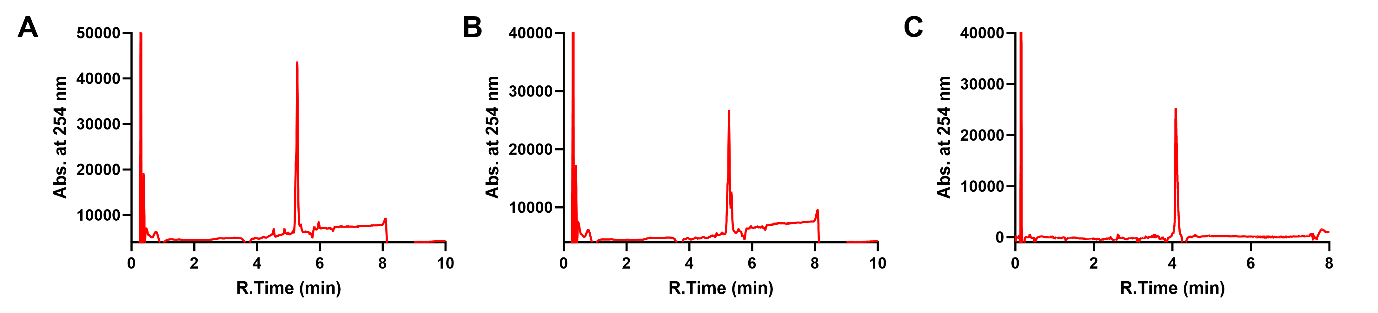


**Figure S48.** HPLC traces for compounds **2** (**A**), **3** (**B**), and **BPcSO_3_** (**C**).

**11 References**

1. S D. J. Peeler, R. Sun, C. Kütahya, P. Peschke, K. Zhou, G. Brachi, J. Yeow, O. Rifaie-Graham, J. P. Wojciechowski, T. F. F. Fernandez Debets, V. LaLone, X. Song, K. Polra, P. F. McKay, J. S. Tregoning, R. J. Shattock, M. M. Stevens, *Adv. Mater.* **2025**, 2417290 [↑](#endnote-ref-2)
2. S a) J. Meinecke, U. Koert, *Org. Lett.* **2019**, *21*, 7609; b) J. G. K. O’Brien, S. R. Chintala, J. M. Fox, *J. Org. Chem.* **2018**, *83*, 7500. [↑](#endnote-ref-3)
3. S J. Dommerholt, S. Schmidt, R. Temming, L. J. A. Hendriks, F. P. J. T. Rutjes, J. C. M. van Hest, D. J. Lefeber, P. Friedl, F. L. van Delft, *Angew. Chem. Int. Ed.* **2010**, *49*, 9422. [↑](#endnote-ref-4)
4. S L. N. C. Rochet, C. Bahou, J. P. Wojciechowski, I. Koutsopetras, P. Britton, R. J. Spears, I. A. Thanasi, B. Shao, L. Zhong, D.-K. Bučar, A. E. Aliev, M. J. Porter, M. M. Stevens, J. R. Baker, V. Chudasama, *Chem. Sci.* **2023**,*14*, 13743. [↑](#endnote-ref-5)
5. S D. Kand, P. Liu, M. X. Navarro, L. J. Fischer, L. Rousso-Noori, D. Friedmann-Morvinski, A. H. Winter, E. W. Miller, R. Weinstain, *J. Am. Chem. Soc.* **2020**, *142*, 4970. [↑](#endnote-ref-6)
6. S P. Shrestha, K. C. Dissanayake, E. J. Gehrmann, C. S. Wijesooriya, A. Mukhopadhyay, E. A. Smith, A. H. Winter, *J. Am. Chem. Soc.* **2020**, *142*, 15505. [↑](#endnote-ref-7)
